# Isolation and characterization of *Plasmodium falciparum* blood-stage persisters by improved selection protocols using dihydroartemisinin alone

**DOI:** 10.1128/aac.00053-24

**Published:** 2025-02-10

**Authors:** Daniel Kiboi, Juliana M. Sá, Akshaykumar Nayak, Chiara E. Micchelli, Shuchi N. Amin, Alexander G. Burbelo, Sasha A. Abielmona, Brian Xi, Lucia A. Mulei, Noah M. Onchieku, Caroline M. Percopo, Jianbing Mu, Thomas E. Wellems

**Affiliations:** 1 Laboratory of Malaria and Vector Research, National Institute of Allergy and Infectious Diseases 550430 , Bethesda, Maryland, USA; 2 Department of Biochemistry, Jomo Kenyatta University of Agriculture and Technology 118985 , Nairobi, Kenya; The Children's Hospital of Philadelphia, Philadelphia, Pennsylvania, USA

**Keywords:** malaria, artemisinin, autophagy, dormancy, drug resistance, recrudescence

## Abstract

Artemisinin-based combination therapies (ACTs) are vital for malaria treatment, but these are threatened by blood-stage persisters—dormant forms of *Plasmodium* parasites that can survive drug exposure and cause recrudescent infections. Here, we present improved protocols for efficient preparation of pure *Plasmodium falciparum* persister populations without the need for magnetically activated columns, sorbitol exposure, or prolonged manipulations. Our protocols transformed actively replicating parasites into persister populations by exposing mixed blood-stage parasites to three or four consecutive daily 6 h pulses of 700 nM or 200 nM dihydroartemisinin (DHA). In micrographs of Giemsa-stained cells, we observed different persister morphologies: Type I persisters containing a rounded magenta-stained nucleus accompanied by a local region of blue-stained cytoplasm; and the more-prevalent Type II persisters characterized by a dark round or irregular-appearing nucleus and faded or no detectable cytoplasm. We also observed cells with disorganized nuclear and cytoplasmic structure, suggesting possible autophagic processes of destruction and remodeling. Recrudescence of actively replicating parasites to starting parasitemia or higher occurred around 17–22 days after initial DHA exposure. Differential expression patterns of the acetyl CoA carboxylase (*acc*) and skeleton binding protein 1 (*sbp1*) genes during DHA treatment, dormancy, and recrudescence highlighted the evolution of physiologic states and metabolic changes underlying persister formation and recovery. Our findings suggest hypotheses and questions for further research to understand the cellular pathways of dormancy and uncover strategies to thwart parasite survival after drug exposure.

## INTRODUCTION

Artemisinin and its semisynthetic derivatives (ART) are essential drugs used worldwide against *Plasmodium falciparum* infections (1), but despite their ability to rapidly clear the parasitemia and symptoms of malaria, recrudescences have been frequently observed after courses of ART monotherapy, even when administered daily for 3 days or longer (2–5). These recrudescences are thought to arise from subpopulations of parasites (persisters) that enter a state of dormancy, circulating in the bloodstream for days or weeks before reactivating to initiate new infections (6–12). The inherent ability of malaria parasites to survive drug exposure through dormancy is supported by observations of recrudescences in primate and rodent *Plasmodium* infections after ART treatment, as well as the lack of association between changes in ART half-maximum inhibitory concentrations (IC_50_s) and observed parasite clearance times or treatment outcomes *in vivo* (13–17). Like in cancer and bacteria research, various terms, such as dormant, quiescent, tolerant or persister cells, have been used to describe these growth-arrested, non-replicating subpopulations that survive drug exposure (8, 18–20). In this study, we refer to these malaria parasites as dormant forms or persisters.

While dormancy has been widely studied in *Plasmodium* parasites exposed to ART, it is not unique to this class of drugs. The concept of *P. falciparum* survival through dormancy was first proposed based on *in vitro* experiments with parasites exposed to sorbitol or pyrimethamine for four consecutive days (21). Subsequent laboratory studies demonstrated dormancy in *P. falciparum* cultures after exposure to ART (7, 22) and other drugs, including mefloquine (23), atovaquone (24), and chloroquine (25). ART-induced dormancy has received particular attention due to the drug’s critical role in malaria treatment and the challenges that persisters pose for artemisinin-based combination therapy (ACT), as they demand effective partner drugs for complete clearance of infections. The clinical significance of dormancy has been underscored by analyses of treatment outcomes and results from animal malaria models, highlighting the importance of effective partner drugs in preventing recrudescences (6, 11, 15, 17, 26).

Recent molecular studies have provided insights into mechanisms underlying parasite dormancy. Cell cycle regulation may play a crucial role in this process, as studies of cyclin-dependent kinases, including cdc2-related protein kinase 1 (*crk1*) and cdc2-related protein kinase 4 (*crk4*), have shown correlations with cell cycle stalling at the G- to S-phase transition during dormancy (27). Metabolic pathways also seem to be involved, including those found in the apicoplast and mitochondrion. Transcriptomic analyses have revealed upregulation of genes encoding key enzymes in fatty acid synthesis and pyruvate metabolism, including pyruvate kinase 2 (*pykii*), a putative elongation of fatty acid homologue (*elo3*), acetyl–CoA carboxylase (*acc*), lipoyl synthase (*lipA*), and biotin–protein ligase 1 (*hcs1*) (10, 28). The importance of *acc* upregulation was further confirmed in a controlled human malaria infection study, in which dormant parasites showed increased expression of this gene 72 hours after treatment with a single oral dose of artesunate (11). Downregulation of most cellular functions associated with growth and development but upregulation of certain other functions and DNA repair, consistent with both cellular quiescence and senescence, were recently reported from transcriptional profiling study of DHA-induced persisters (29). Additionally, skeleton-binding protein 1 (*sbp1*), a component of the Maurer’s cleft (30–32), has been associated with parasite persistence in patients treated with ART, as well as non-ART drugs (33–35). Notably, *sbp1* transcripts are primarily found in the asexual ring stage (36–38), further supporting the hypothesis that this early stage may be particularly prone to entering dormancy.

Various laboratory protocols have been employed to study the dormancy and recrudescence of *P. falciparum in vitro* (7, 10, 27, 28, 39, 40). A common approach employs a single 6 h exposure of synchronized, early ring-stage parasites to 700 nM dihydroartemisinin (DHA). However, two issues can arise with this method as usually implemented. First, to obtain highly synchronous parasites (*ca*. 95%), two rounds of sorbitol treatments are typically performed 2 days before the start of DHA exposure to ensure that predominantly <6 h-old ring stages receive the DHA pulse (10, 27, 28, 40, 41). A previous study showed sorbitol itself can select dormant parasites (21), which may confound the interpretation of experiments designed to study dormancy after exposure to DHA or other compounds. The second issue is the need to be sure no active-stage parasites remain that may not have become dormant or killed by the single 6 h DHA pulse. Mature active stages can be removed by passing the culture through magnetic-activated cell sorting columns (MACS, Miltenyi Biotec) or by sorbitol treatments for three consecutive days after the DHA exposure (7, 22, 39). However, as the MACS columns are expensive, and both MACS and sorbitol treatment protocols are time-consuming and cumbersome, improved methods are desirable for the efficient preparation and study of dormant parasites.

Multiple daily DHA pulses on unsynchronized parasite cultures offer an alternative to the above protocols. In a previous study, Teuscher et al. (7) examined the recovery rates of dormant parasites after three daily 6 h 700 nM DHA treatments without MACS column purifications. However, sorbitol synchronization was still applied 2 days before the first DHA treatment. In drug susceptibility experiments on dormant parasites, Reyser et al. (42) initiated quiescence using a single 6 h 700 nM DHA pulse and then continued exposure of the parasites to 700 nM DHA plus various test compounds for an additional 48 h. Building on these approaches, we present efficient and easily performed protocols for the routine production of dormant *P. falciparum* parasites without the need for sorbitol treatments or MACS column purifications. Using these protocols with three or four consecutive daily 6 h exposures of 200–700 nM DHA, we show how persister populations can be purified by fluorescence-activated cell sorting (FACS), characterized by cellular studies, and analyzed for molecular processes potentially involved in dormancy and recrudescence. Our results suggest hypotheses and questions for further research to understand the persister state, illuminate the cellular and signaling events of dormancy, and uncover strategies to thwart parasite survival after drug exposure.

## RESULTS

### Recrudescences from unsynchronized parasites after treatment with multiple daily pulses of DHA

To develop a protocol for routine and inexpensive production of *P. falciparum* persisters without the need for synchronization, column purifications, or sorbitol treatment after DHA exposure, we subjected asynchronous *P. falciparum* cultures of GB4 and 803 parasites (1.5–2% initial parasitemia) to daily 6 h pulses of 700 or 200 nM DHA for four consecutive days (4DP protocol). This 4DP treatment period spanned the equivalent of two complete asexual cycles of untreated parasites (Fig. S1). The 700 nM DHA concentration (200 ng/mL) was based on its use to induce dormancy in previous studies (7, 28, 39). The 200 nM concentration (57 ng/mL) was chosen to represent a level in the 6 h range of the smaller peaks of plasma DHA reported after four-tablet doses of artemether (80 mg total) + lumefantrine (480 mg total) in Thai patients (43). After each pulse, the cells were pelleted by centrifugation, washed twice in culture medium, and immediately returned to culture. No sorbitol exposure was applied to the parasites prior to or during the experiments.

**Fig 1:**
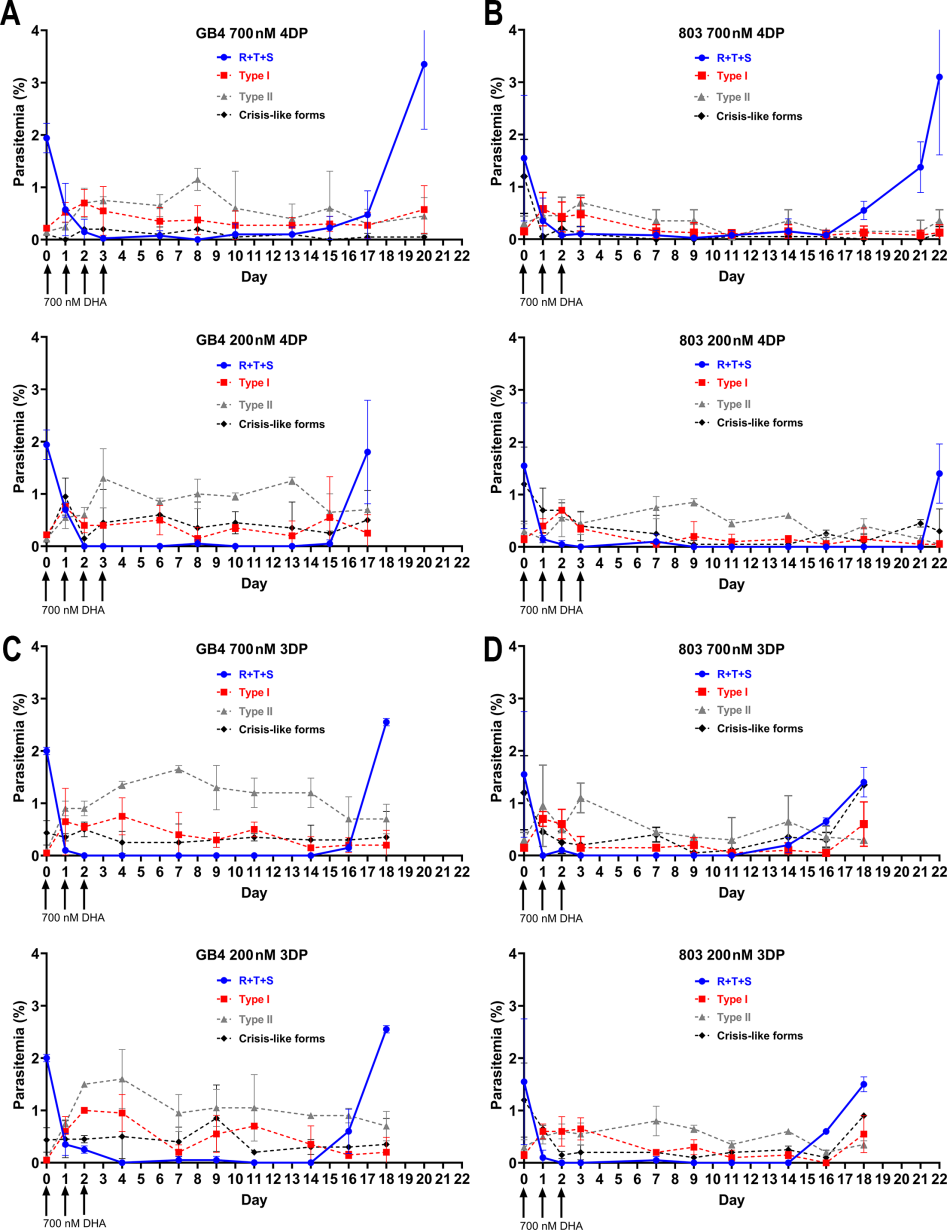
Recrudescence assay results from asynchronous cultures of *P. falciparum* parasites exposed to daily 6 h pulses of dihydroartemisinin (DHA). Pulses of 700 or 200 nM DHA were given on the days indicated by the arrows. (**A**) Results from *P. falciparum* line GB4, which contains no mutations in the PfK13 propeller region, exposed to four 6 h daily pulses of 700 or 200 nM DHA (4DP protocol). (**B**) 4DP results from *P. falciparum* line 803, which contains the C580Y mutation in the PfK13 propeller region. (**C**) Results from *P. falciparum* line GB4 exposed to three 6 h daily pulses of 700 or 200 nM DHA (3DP protocol). (**D**) 3DP results from *P. falciparum* line 803. Parasitemia percentages of actively growing blood stage forms (RTS; rings, trophozoites, and schizonts) are indicated by solid blue lines. Parasitemia percentages of Type I and II persisters are represented by the dashed red and gray lines, respectively, and those of crisis-like forms by the dashed black lines. No gametocytes were observed in these experiments. Counts for analysis were recorded from each Giemsa-stained thin blood film by at least two microscopists blinded to slide identification. Points and error bars represent the means and standard deviations.

Following the first daily pulse of DHA, we observed rapid reductions of actively replicating ring, trophozoite, and schizont (RTS) stages (Fig. 1A and B) in agreement with previous results from *P. falciparum* lines GB4 and 803 and other parasite clones (17, 39, 40, 44). In concert with this decline, persisters quickly rose in the counts and remained present in culture for the duration of the experiment. Using Giemsa dye for microscopy visualization, we classified these persisters according to the morphologies described by Tucker et al. (45): dormant-like persisters (hereafter referred to as Type I) were identified by a small, rounded magenta-stained nucleus and accompanying blue-stained cytoplasm; and pyknotic-like persisters (Type II) contained a dark round or irregular-appearing nucleus accompanied by faded or no detectable cytoplasm. Type II persisters consistently outnumbered the Type I persisters in our data. Whether treated with 700 or 200 nM DHA, the GB4 and 803 parasites in these 4DP assays recrudesced to starting parasitemia levels or higher 17–22 days after the first dose of DHA (Fig. 1A and B). Among the persisters, we also observed variable numbers of parasites with degenerative changes resembling crisis forms (46–48).

Having characterized the recrudescences and persisters from 4DP assays with 700 nM DHA, we investigated whether similar results could be obtained from three daily 6 h drug pulses (3DP protocol) applied to asynchronous parasitized red blood cells (pRBC) from *P. falciparum* cultures. This program of 3DP exposure aligns with the 3-day period typical of artemisinin-based combination therapy (ACT) used for malaria treatment. After we exposed GB4 and 803 parasites to the three daily 6 h pulses of 700 or 200 nM DHA, we found rapid reductions of actively replicating RTS accompanied by the appearance of Type I and II persisters as in the 4DP assays (Fig. 1C and D). Recrudescences above the level of the starting parasitemia percentage was observed around day 18. These results suggest that 3DP was as effective as the 4DP protocol for clinically relevant studies of ART-induced dormancy and ACT partner drug resistance.

### Flow cytometry, purification, and characterization of ART-treated parasites from recrudescence assays

The persisters in these experiments were detected by flow cytometry of cells doubly stained with SYBR Green I (SG, nucleic acid dye) and MitoTracker Deep Red (MT, mitochondrial membrane potential dye). In MACSQuant Analyzer displays of GB4 and 803 parasites exposed to 4DP (700 nM), persisters presented as a clustered population above and slightly to the right of the center of uninfected erythrocytes (uRBC) due to their SG and MT fluorescence (Fig. S3A and B). Between days 2 and 15, the parasitemia percentages in these clusters ranged from 1.31 to 2.55% for GB4 and 2.06 to 2.91% for 803 parasites. Compared to the clusters of persisters during dormancy, the distributions of recrudescing RTS populations showed rightward and upward extensions of more brightly fluorescing SG^+^/MT^+^ cells. This pattern is consistent with the greater DNA content and mitochondrial activity of the trophozoite and schizont stages (Fig. S3A, days 17 and 20, and asynchronous control panel; Fig. S3B, days 14–18 and asynchronous control panel).

**Fig 2:**
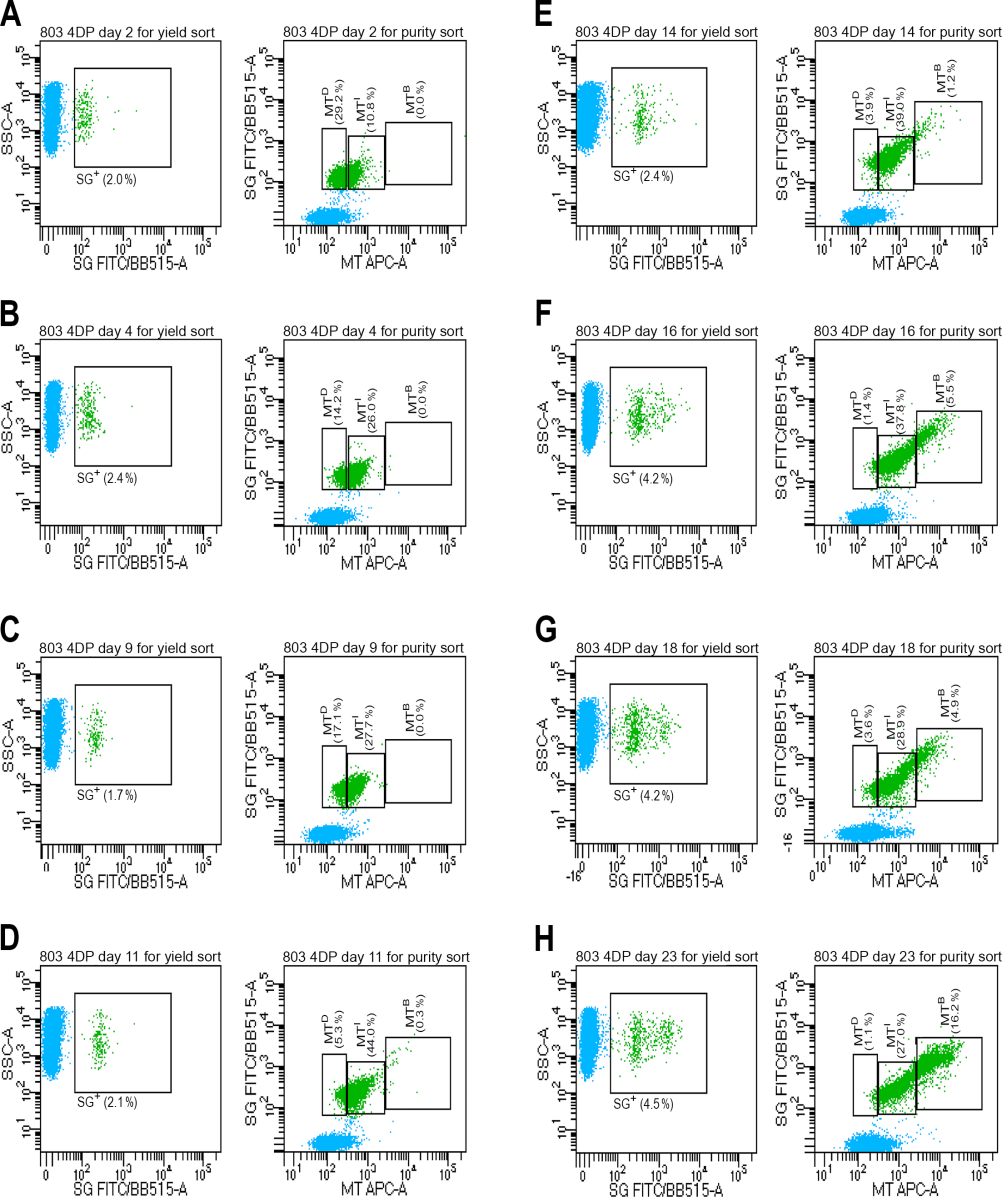
Fluorescence-activated cell sorting (FACS) results from a 4DP assay of *Plasmodium falciparum* 803 parasites. An unsynchronized culture at 2% initial parasitemia was treated with 700 nM DHA for 6 h on days 0, 1, 2, and 3. Cells from culture samples were stained with SYBR Green I (SG) and MitoTracker Deep Red (MT) for FACS analysis and purification on days 2, 4, 9, 11, 14, 16, 18, and 23. (A–H) Left and right panels show the parasite distributions and gates for the two steps of FACS purification by a BD Aria fusion flow cytometer. Left panels indicate SYBR Green I (SG) and Side Scatter (SS) levels of SG^+^ pRBC subjected to yield sorting. Right panels show the three gates delineating the MT dim (MT^D^), MT intermediate (MT^I^), and MT bright (MT^B^) assigned to the yield-sorted pRBC for final purity sorting.

To further study the changes accompanying *P. falciparum* persistence and recrudescence, we used FACS to isolate and analyze purified populations of pRBC (803 line) at days 2, 4, 9, 11, 14, 16, 18, and 23 of 4DP with 700 nM DHA. A BD Aria Fusion flow cytometer was employed in a two-step process using yield mode for initial enrichment, followed by four-way purity mode for precise separation of parasite populations. This approach allowed us to isolate and collect cells from samples stained with SG and MT. In the first yield-sorting step, samples were assessed for SG fluorescence, and all SG^+^ cells (including persisters and any RTS stages) were collected by high-throughput gating (Fig. 2A through H, left panels). The parasitemia percentages in this SG^+^ gate were consistent with the frequencies of persisters plus RTS stages estimated from Giemsa-stained thin films (Fig. 1B) or MACSQuant data (Fig. S3B).

Prior to the second step of purity sorting, FACS analysis of the SG^+^/MT^+^ cells obtained by yield-sorting showed that most uRBC had been removed from each sample, effectively enriching the pRBC population (Fig. 2A through H, right panels). We assigned three gates to these pRBC for purity-sort collection and further analysis:

1. MT^D^ for pRBC with dim MT signals overlapping with the upper range of uRBC background fluorescence;
2. MT^I^ for pRBC with intermediate MT signals extending from the upper boundary of uRBC fluorescence, including the P2 and P3 regions of persister and ring-stage fluorescence delineated by Micchelli et al. (44).
3. MT^B^ for pRBC defined with bright fluorescence characteristic of trophozoites and schizonts.

In agreement with the findings from Giemsa-stained films and MACSQuant analysis (Fig. 1; Fig. S3), signals from persister cells on days 2–11 dominated in the MT^D^ and MT^I^ gates, with few or no signals detected in the MT^B^ gate (Fig. 2A through D, right panels). Beginning on day 11, we observed markedly decreased signals in the MT^D^ gate relative to the steadier signal rate in the MT^I^ gate and rising rate in MT^B^ gate (Fig. 2D through H, right panels). The decreases in the MT^D^ gate and increases in the MT^B^ gate from days 11 to 23 are consistent with declining numbers of persisters and concomitantly expanding populations of recrudescent RTS observed by microscopy (Fig. 1).

**Fig 3:**
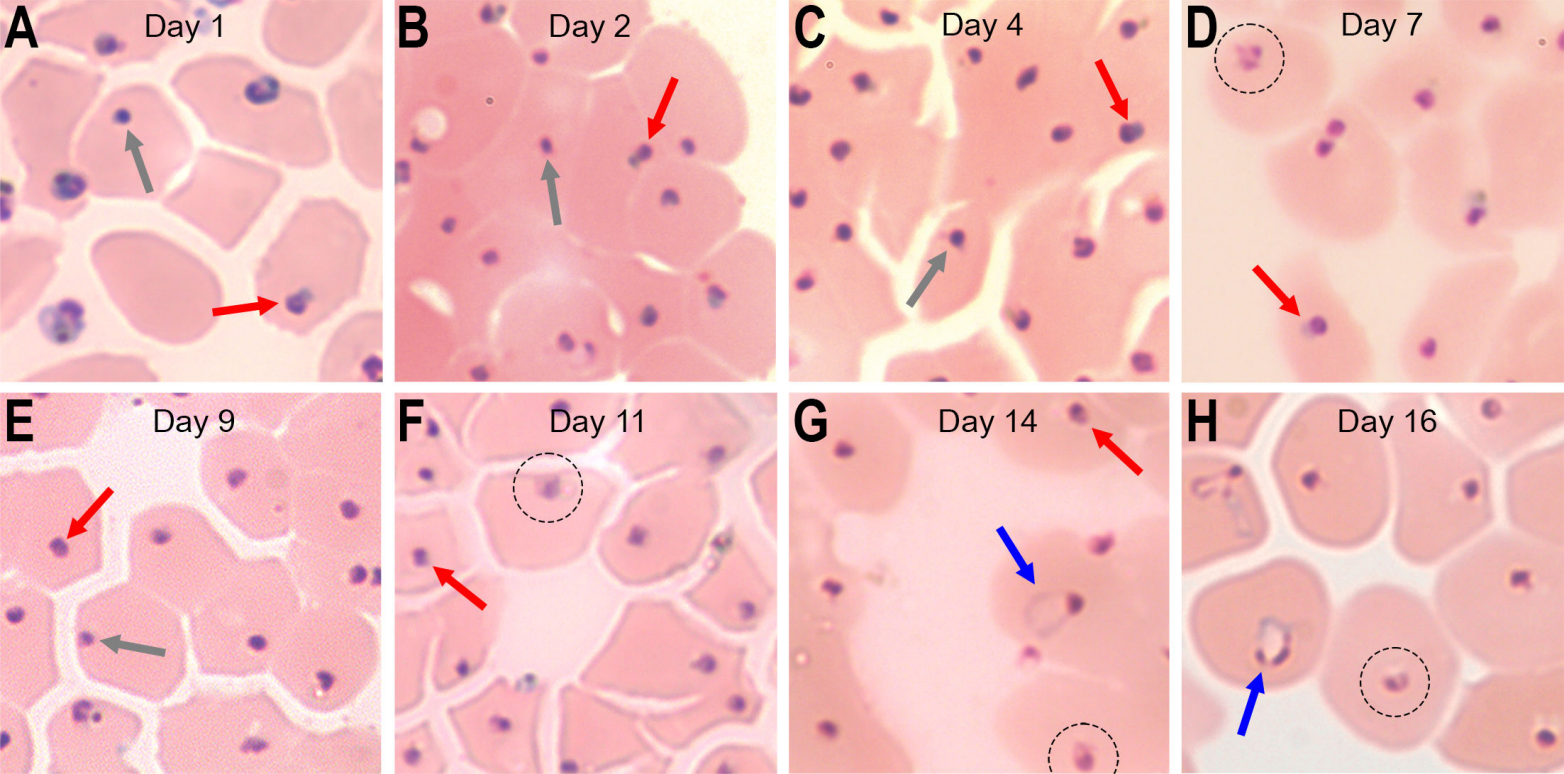
Giemsa-stained images of FACS-collected SG^+^/ MT^I^ *P. falciparum* 803 parasites selected by the 4DP protocol. The images are from Giemsa-stained monolayers obtained after Cytospin centrifugation. A range of persister forms is apparent, including Type I forms with a small, rounded nucleus and apparent cytoplasm (red arrows); and Type II forms with a rounded, intensely stained nucleus but little stained cytoplasm (gray arrows) (45). (**A**) Abundant large irregular parasites with crisis-like appearance are found in cytospin preparations on day 1. (**D, F, G, H**) Examples of cells appearing to have disorganized structures suggestive of autophagy are circled. (**F, G**) Recrudescent ring stages are indicated by blue arrows. Additional images from Giemsa-stained thin blood films are shown in Fig. S2.

Purity-sorted pRBC from the BD Aria Fusion flow cytometer were centrifuged onto microscope slides in a Cytospin 4 cytocentrifuge using EprediaCytofunnels fixed with 100% methanol and Giemsa-stained for microscopy. Fig. 3A through H presents micrographs of the MT^I^ parasites collected from 803 4DP, 700 nM DHA recrudescence assays at days 1, 2, 4, 7, 9, 11, 14, and 16. In these images, we observed persister forms, including types I and II but no circular-shaped ring forms until day 14 (Fig. 3G). This finding of ring forms on day 14 was consistent with the rise of MT^B^ parasites detected by FACS analysis (Fig. 2E). Intraerythrocytic parasites with disorganized structures were often found during the second and third weeks of the assay (Fig. 3D, F, G and H), consistent with the apicoplast heterogeneity and possible autophagy suggested in our previous study DHA/sorbitol-induced persisters (44).

### Transcription levels from genes encoding acetyl CoA carboxylase and skeleton-binding protein 1

Genes involved in fatty acid synthesis and pyruvate metabolism remain active after ART-induced dormancy in *P. falciparum*, suggesting their expression is vital to persister viability (10, 28). Chen et al. (28) examined the transcripts of genes, including PF3D7_1026900 (NCBI gene ID 8445054, identified as “*acc*” in Ref. [28] but encoding the biotin–protein ligase one paralog PfHCS1 [49]) and PF3D7_1469600 (NCBI gene ID 812246, encoding acetyl–CoA carboxylase with a sequence named *bc* in Refs. [28] and [11]). Results of that study showed that a single 6 h pulse of DHA increased transcription of PF3D7_1026900 and PF3D7_1469600 by 1.83- and 3.72-fold on day 2, respectively, after which transcription of these genes returned to baseline and lower levels on days 3–8.

Here, we follow the conventions of Goodman et al. (50) and Dellibovi-Ragheb et al. (49) and refer to the PF3D7_1469600 acetyl–CoA carboxylase gene as *acc*. Using qRT-PCR with the primers and probe identified in Fig. S4 and Table S1, we compared the time course and levels of *acc* upregulation after DHA induction with a single 6 h pulse (1DP protocol). Results from this 1DP protocol showed *acc*/PF3D7_1469600 transcription increased by 2.88-fold at 6 h and 4.89-fold at 48 h post-treatment (Fig. S5A).

**Fig 4:**
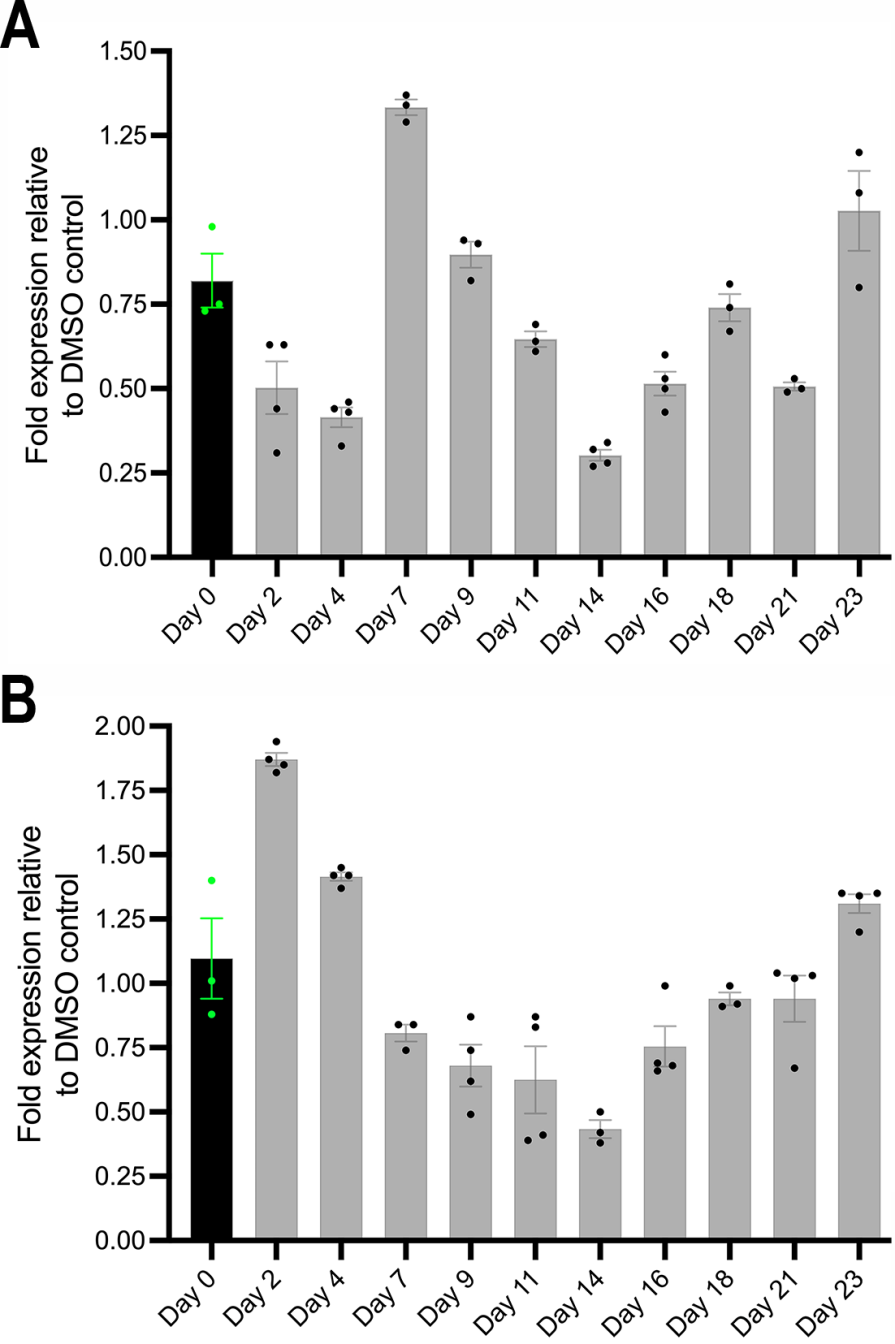
Relative levels of acetyl–CoA carboxylase (*acc*) and skeleton-binding protein 1 (*sbp1*) transcripts after 4DP exposures of *P. falciparum* 803 parasites to 700 nM dihydroartemisinin. (**A**) Relative levels of reverse-transcribed *acc* transcripts. (**B**) Relative levels of reverse-transcribed *sbp1* transcripts. qRT-PCR measurements were performed at the initiation of DHA treatment on day 0 (black vertical bar) and at indicated days thereafter (gray vertical bars). Data points represent means from three independent replicates, with error bars indicating standard deviations.

Next, we assessed *acc* transcript levels during and after four daily 6 h pulses of 700 nM DHA in 4DP recrudescence assays. These confirmed a dynamic pattern of *acc* expression through dormancy and into recrudescence (Fig. 4A). Initially, *acc* transcript levels decreased relative to untreated controls (0.6-fold on day 2, *P* = 0.03; 0.5-fold on day 4, *P* = 0.03). However, an upregulation occurred on day 7 (1.6-fold, *P* = 0.05), followed by a return to near-control levels on day 9 (1.1-fold; *P* = 0.23). After further decreases on days 11 and 14 (0.8-fold, *P* = 0.05 and 0.4-fold, *P* = 0.03, respectively), the *acc* transcript levels rose again (0.6-fold on day 16, *P* = 0.03; 0.9-fold on day 18, *P* = 0.35). After a dip at day 21 (0.6-fold, *P* = 0.05), the *acc* transcript levels returned to those of control parasites on day 23 (1.3-fold, *P* = 0.1).

We also evaluated transcript levels of *sbp1*, as expression of this gene after ACT treatment (36–38) may be consistent with a role in persister viability and replication upon recrudescence. After a single 6 h 700 nM DHA pulse in the 1DP protocol, *sbp1* was upregulated at 6 h (2.1-fold, *P* = 0.05) and 48 h (1.9-fold, *P* = 0.05) (Fig. S5B). In the 4DP protocol, *sbp1* transcript levels on day 2 were increased 1.7-fold relative to the pretreatment level (*P =* 0.04) (Fig. 4B). The ratios then showed a declining trend to 1.3-fold on day 4 (*P =* 0.06), 0.7-fold on day 7 (*P* = 0.05), reaching 0.4-fold on day 14 (*P* = 0.03) before returning to near those of control parasites [0.9-fold on day 18, (*P =* 0.35); 1.2-fold on day 23, (*P =* 1.2)].

## DISCUSSION

Exposing asynchronous *P. falciparum* cultures to multiple daily 6 h pulses of DHA provides a robust and efficient means for the production and study of dormant persisters. In the 3DP, as well as 4DP, protocols, actively replicating parasites were reliably transformed into persister populations when exposed to 200 or 700 nM DHA. Type II persisters characterized in Giemsa-stained cells by dark round or irregular-appearing nuclei and faded or no detectable cytoplasm, dominated in these populations, while Type I persisters containing rounded nuclei and readily detected cytoplasm were less frequently observed. Importantly, recrudescence of actively replicating parasites was consistently obtained in our protocols, with rings appearing in Cytospin preparations around 2 weeks after the initial DHA exposure. Using persisters purified by FACS after SG and MT staining, we found that transcripts from genes encoding *acc* and *sbp1* were differentially expressed along the timeline of persister induction, dormancy, and recrudescence, reflecting the metabolic changes enabling parasite survival and recovery (28). These findings highlight the remarkable ability of malaria parasites to survive drug exposure. Multi-DHA-pulse protocols will facilitate insights into the physiological states of persisters and the molecular processes of dormancy and recrudescence, potentially suggesting new strategies for drug development.

Protocols applying a single 6 h DHA pulse to synchronized rings typically use several daily rounds of follow-up magnetic column purification or sorbitol lysis to ensure the elimination of actively replicating parasites (7, 10, 28, 39, 40). While effective, these methods have potential drawbacks. MACS column purification is costly and subjects cells to prolonged stress outside of culture conditions. Sorbitol treatments for parasite synchronization and/or follow-up removal of actively replicating parasites involve multiple manipulations that can induce dormancy independently (21), potentially confounding studies of dormancy induced by ART and other antimalarial drugs. In contrast, our 3DP and 4DP protocols, by applying multiple daily 200 or 700 nM DHA pulses to mixed parasite stages, transform and eliminate the actively replicating parasites without additional manipulations. This approach simplifies the prodecure, reduces potential confounding factors, and enhances the reliability of preparations for investigations of persister biology and drug resistance mechanisms.

Our study examined transcript levels from two key genes, acetyl CoA carboxylase (*acc*) and skeleton binding protein 1 (*sbp1*), which are implicated in persister viability after ART exposure (28, 33–35). Consistent with the report of Chen et al. (28), our 1DP protocol using a single 6 h DHA exposure showed 2.88-fold *acc* upregulation at 6 h and 4.89-fold upregulation at 48 h. However, with the 4DP protocol, we found 0.5–0.6-fold downregulation of *acc* transcript levels through day 4, then a bounce to 1.6-fold upregulation at day 7, before a steady decline to 0.3-fold at day 14. The use of four daily DHA treatments in the 4DP protocol may be responsible for later upregulation of *acc* transcription (on day 7) compared to the upregulation seen after the single DHA treatment in the 1DP protocol (on day 2). In both cases, *acc* upregulation after the completion of DHA exposure points to an important role of fatty acid synthesis in persister development before transcription falls to lower levels later in dormancy (28). The later day-7 peak of *acc* transcription after four daily DHA pulses compared to the day-2 peak after the single DHA pulse suggests that time course of dormancy induction can depend upon the number of drug pulses. Thus, depending upon the induction protocol, persister development or ‘maturation’ may extend beyond the 5-day period that has been proposed after a single DHA pulse (29).

Transcription levels of *sbp1*, a gene crucial for protein export and cytoadherence (51, 52), also exhibited dynamic changes during our 4DP experiments. Notably, *sbp1* transcripts were detected during days 2–9 when no active parasite stages were observed, suggesting origin of these transcripts from persisters. This result aligns with the continuing presence of *sbp1* signals found after ACT treatment of *P. falciparum* infections in clinical studies (33, 35). Distinct transcription patterns of *acc* and *sbp1* highlight the complex metabolic and cellular adaptations of persisters during dormancy and recovery.

In FACS preparations, MT^+^/SG^+^ fluorescence was detected from both type I and II persisters throughout the dormancy period, indicating they maintained mitochondrial membrane potential, a key indicator of cellular viability, despite their morphological differences in Giemsa-stained thin film preparations. These findings complement super-resolution microscopy evidence showing that MT^+^ mitochondria in the persisters enlarge and move into closer association with nuclei, potentially participating in retrograde signaling responses to stress (40, 44). Our observation of energetically functional mitochondria lasting for weeks in the whole SG^+^ population challenges the notion that a large fraction of Type II/pyknotic-like persisters is dead. Instead, this evidence for intact mitochondria in the SG^+^ population suggests that some metabolic processes may be sustained in Type II persisters during the dormancy period.

These experiments in this report were not designed to look for differences of time-to-recrudescence due to parasite genetic backgrounds, DHA exposure number in the 4DP and 3DP protocols, or other variations from factors such as shelf age or blood types of erythrocytes from different donors. In a previous study of PfK13 mutations and laboratory measures for ART resistance, we crossed the GB4 (PfK13 wild type) and 803 (carrying the PfK13 C580Y mutation) lines and examined the inherited genotypes and phenotypes of the parents and progeny (17). Although ring-stage survival assays (RSAs) of the parents and progeny confirmed linkage of the C580Y mutation to proportionally greater survival of ring stages to a 6 h pulse of 700 nM DHA, similar low nM IC_50_ levels were found for all the parasites after exposure to DHA for 72 h. Data from artesunate-treated New World monkeys also showed no significant differences in the parasitemia clearance half-lives or recrudescences of the *in vivo* infections from the parasites carrying either a wild-type or C580Y-mutant PfK13. Further experiments were then performed to test for an effect of the C580Y on recrudescence *in vitro* using an isogenic pair of clones engineered to differ only by this mutation (76H10^580Y^ and 76H10^C580^). In replicate head-to-head comparisons, recrudescences of the 76H10^580Y^ parasites (nominally ‘resistant’ by RSAs) occurred no sooner than those of the isogenic 76H10^C580^ parasites (‘sensitive’ by RSAs; see supplemental Fig. S8 of Sá et al. [17]). These findings agreed with those of Breglio et al. (39) who examined a different set of isogenic lines and found no association of Kelch mutations C580Y or R539T to *in vitro* recrudescence times, whereas parasites of different backgrounds showed differences suggesting possible effects from other, perhaps multiple genetic determinants.

In a recent study of persisters after DHA/sorbitol treatment (44), we found progressive heterogeneity of enlarged and irregularly shaped apicoplasts as the persisters aged. These results suggested that apicoplasts may be subject to autophagic processes analogous to the processes of chlorophagy found after chloroplast swelling, vesiculation, and degradation from ROS damage in plants (53, 54). Here, we provide two additional findings that lend further credence to the suggestion of autophagy in persisters. First, Giemsa-stained images of Type II persisters in the MT^+^ populations purified by FACS showed minimal (“faded”) or no stained cytoplasm. This lack of stained cytoplasm in Type II images agrees with findings of “vacant cytoplasm” associated with enlarged mitochondria, reduced ribosomes, and multi-membrane structures in the transmission electron micrographs of persisters reported by Tripathi et al. (29). Second, cells with disorganized structure were present in our Cytospin preparations of MT^+^ persisters at the second and third weeks of dormancy. The MT positivity of these cells is more consistent with autophagy than with cell death by primary apoptosis or necrosis as these latter two processes typically show early loss of mitochondrial function (55).

Autophagy has been previously implicated in *P. falciparum* blood stages stressed by nutrient starvation or exposure to various toxins and drugs, including chloroquine (56–59). While features of apoptosis or necrosis have been proposed from other studies of cell death phenotypes (60), autophagy may be key to the interior cellular remodelings necessary for metamorphosis of *P. falciparum* into various stages, such as gametocytes and liver stage parasites (61, 62). Moreover, the autophagy homolog PfATG8 is vital to apicoplast formation and has been suggested to have roles in both degradative and biosynthetic processes in the cell (59, 63–65). Given these observations and the various non-autophagic roles of ATG-related proteins (66), we speculate that autophagy or autophagic-like processes may be involved in *P. falciparum* persister survival and viability under stress. As in the metamorphosis of other *P. falciparum* stages, autophagic-like processes may also facilitate the remodeling events necessary to transform blood-stage parasites into persisters and later return these persisters to actively replicating forms for recrudescence.

While multiple studies have implicated persisters in recrudescence after DHA-induced dormancy, the origin of actively replicating recrudescent parasites remains to be definitively demonstrated. Recrudescence in culture showed little or no delay after persister populations were exposed for 1 day to pyrimethamine (1–100× IC_50_), making it seem unlikely that a low number of ring stages remains active through the period of DHA treatment and exponentially replicates to produce the recrudescent population (29, 67). Caveats to this conclusion include: the growth of rings and trophozoites was found to be unaffected when treated with 100× IC_50_ pyrimethamine concentrations for 24 h (68); and asynchronous *P. falciparum* cultures predominantly containing ring stages required 53–55 h to effectively clear when exposed to daily concentrations of 3×, 10×, or 100× IC_50_ pyrimethamine (69). In other studies, various antimalarial compounds have been shown to delay recrudescences *in vitro*, suggesting these compounds can remain partially toxic to persisters despite their relatively quiescent state (29, 42, 67). Findings of persisters after ART treatment in mice infected with *P. vinckei* (15), humans infected with *P. falciparum* (11), *Aotus* monkeys infected with *P. falciparum* (17), and *Saimiri boliviensis* monkeys infected with *P. vivax* (Fig. S6) provide further support for the role of these parasite forms in recrudescent infections. In all of these observations, the persisters have featured Giemsa-stained cytoplasm characteristic of Type I morphology. Cytoplasm that has not been hollowed out by autophagy may thus be necessary to power persister remodeling and transformation. Confirmation of this hypothesis may be feasible by isolating the different types of persister stages (I vs. II) and directly studying them for autophagy and reactivation into replicating stages.

The protocols described in this report offer robust and efficient means to prepare pure *P. falciparum* persister populations induced by ART without the expense of magnetic columns, exposure to sorbitol, or prolonged manipulations of previous approaches. These protocols will be valuable for investigating the molecular mechanisms underlying ART-induced dormancy and parasite recrudescence. The evidence for mitochondrial activity and apicoplast heterogeneity in persisters, along with the observed patterns of *acc* and *sbp1* expression, highlight the complex metabolic and structural adaptations underlying the generation of dormant forms and their reactivation. Autophagic-like processes may play a vital role both in maintaining persister viability and facilitating parasite remodeling for dormancy and recrudescence. Future investigations employing methods, such as RNA sequencing and metabolomics, on purified type I and II persister populations will help to elucidate the pathways by which parasites enter dormancy, maintain the dormant state, and reactivate to normal proliferation. These insights will help to identify novel targets and suggest therapeutics to interrupt these processes, potentially preventing parasite recrudescence. By enhancing our understanding of persister biology, this research will lay the foundation for more effective strategies to combat ACT failures and improve malaria treatment outcomes.

## MATERIALS AND METHODS

### *Plasmodium falciparum* parasites, culture maintenance, and microscopy of Giemsa-stained thin films

*P. falciparum* 803 from Cambodia (17, 70) and GB4 from Ghana (71, 72) were cultured in leucocyte-depleted human O^+^ erythrocytes (RBC; Interstate Blood Bank, Inc., Memphis, Tennessee) in complete RPMI (cRPMI) medium. The cRPMI consisted of RPMI 1640 medium (KD Medical, Cat. No. CUS-0645, Columbia, MD) supplemented with 25 mM HEPES, 50 µg/mL hypoxanthine, 0.21% NaHCO_3_ (KD Medical, Cat. No. RGC-5120, Columbia, MD), 20 µg/mL gentamycin (KD Medical, Cat. No. CAG-1240, Columbia, MD), and 1% (wt/vol) AlbuMAX II (Gibco Cat. no 11021-045). Parasite cultures were maintained at 5% hematocrit at 37°C under an atmosphere of 90% nitrogen, 5% oxygen, and 5% carbon dioxide.

For microscopy, thin films of the cells were prepared on glass slides, fixed with 100% methanol for 5 s, and air-dried in a dust-free environment. The slides were then stained by immersion for 30 min in a 25% Giemsa solution (Sigma-Aldrich, St. Louis, MO) prepared using Milli-Q purified water (Millipore Sigma, USA). After staining, the slides were rinsed with Milli-Q water and air-dried before microscopic examination using an Olympus CX41 microscope with a 100× oil immersion objective. Images of the stained cells were acquired using a camera and Infinity Analyze Software (ACCU-SCOPE version 7.1.0). Two to four experienced microscopists blinded to slide identification independently assessed 1,000 RBCs from each thin film for the percentage calculations of rings, trophozoites, schizonts, crisis-like forms, and type I or II persisters. Statistical analyses of the parasitemia counts were performed using GraphPad Prism 9 software (GraphPad Software, San Diego, CA).

### Recrudescences and sample collection from DHA-treated parasites *in vitro*

Unsynchronized parasites were established in 10 or 15 mL culture volumes at 2% parasitemia and 5% hematocrit. These were exposed to 6 h daily pulses of dihydroartemisinin (DHA; ≥97% (TLC) purity, Sigma Aldrich, Cat. No. D7439, USA) at concentrations of 200, 400, or 700 nM in cRPMI. DHA stock solutions were prepared in dimethyl sulfoxide (DMSO; ≥99.9% purity, Sigma Aldrich, Cat. No. 276855, USA) and diluted in cRPMI such that the final medium contained ≤0.1% residual DMSO. For each experiment, a parallel 15 mL culture was established from the same master culture and exposed to 0.1% DMSO without DHA as a vehicle control.

After each 6 h DHA or vehicle control exposure, the cells were washed twice with 10 or 15 mL of pre-warmed cRPMI, centrifuged at 2,500 rpm for 5 min in a bench-top centrifuge (Eppendorf Centrifuge 5810) at room temperature, resuspended in 10 or 15 mL cRPMI, and returned to culture. To maintain hematocrit and prevent potential hemolysis, 100 µL of freshly washed RBC [50% hematocrit in incomplete RPMI (iRPMI)] medium were added every 7 days. The iRPMI was composed of RPMI 1640 medium (KD Medical, Cat. No. CUS-0645, Columbia, MD) supplemented with 25 mM HEPES, 50 µg/mL hypoxanthine, 0.21% NaHCO_3_ (KD Medical, Cat. No. RGC-5120, Columbia, MD), and 20 µg/mL gentamycin (KD Medical, Cat. No. CAG-1240, Columbia, MD). On selected days (indicated in the Results section), 100 µL samples from each culture suspension were collected for parasite counts from Giemsa-stained thin smears and flow cytometry analysis; 1 mL samples were used for fluorescence-activated cell sorting (FACS) collections as described below. Culture medium was changed daily during DHA treatment and every 2 days thereafter. Vehicle control cultures containing actively replicating parasites were diluted 1:10 when parasitemia exceeded 2%

### Flow cytometry analysis

A 100 µL volume from each culture was centrifuged at 2,500 rpm for 30 s in a bench-top centrifuge (Eppendorf model 5417R) at room temperature, and the pelleted cells were resuspended to 190 µL in wash buffer (1× Hanks’ balanced salt solution; Gibco, Cat. No. 14025092, Waltham, MA) containing 2% fetal bovine serum (Gibco, Cat. No. 26140079, Waltham, MA). To stain the parasitized erythrocytes (pRBC), 8 µL of stock SYBR Green I (5 × SG from 10,000× concentrate in DMSO; Invitrogen, Cat. no S7585, USA) and 2 µL of stock 7.5 µM MitoTracker Deep Red (MT, Thermo Fischer Scientific, Cat. no M22426, USA) were added to the 190 µL suspension, resulting in final concentrations of 0.2× SG and 75 nM MT. The suspension was then incubated at 37°C for 30 min in the dark. After staining, the cells were washed twice with wash buffer with centrifugation at 2,500 rpm for 3 min in a bench-top centrifuge (Beckman Coulter model Allegra x-14R). The pellet was resuspended in 500 µL of wash buffer for flow cytometry using a MACSQuant Analyzer model 10 (Miltenyi Biotec) using the 488 nm laser excitation and a 525/50 nm bandpass filter for SG fluorescence emission detection and 640 nm excitation and a 665–730 nm bandpass filter for MT fluorescence detection. Photomultiplier tube voltages were set to 350 V for forward scatter, 300 V for side scatter, 500 V for SG, and 450 V for MT.

Unstained and stained uninfected erythrocytes (uRBC) were used for calibration and compensation. At least 1 million cells per sample were counted to quantify the fluorescent cells. Flow cytometer data were processed using MACSQuantify Software (version 2.13.3, Miltenyi Biotec).

### Fluorescence-activated cell sorting (FACS) and collection of SG^+^/MT^+^ cells

Cells were collected from 1 mL volumes of culture suspensions, washed twice with wash buffer, resuspended to 190 µL, and stained with SG and MT, as described above. An Aria Fusion flow cytometer (BD Biosciences) was used to obtain data from at least 1 million events. Analysis was performed with BD FACSDiva Software 9.0 (BD Biosciences, San Jose, CA). Cells were sorted in two steps as recommended by BD Biosciences. In the first step, high-throughput yield sorting for SG^+^ pRBC was performed with 488 nm laser excitation and detection of fluorescence emission with a 530/30 nm bandpass filter. In the second step, the enriched SG^+^ pRBC from the first step were analyzed and further separated by purity sorting using both the 488 nm laser excitation and detection of fluorescence emission with a 530/30 nm bandpass filter, as well as the 637 nm laser excitation and detection of MT fluorescence emission at 670/30 nm bandpass filter. The SG^+^/MT^+^ pRBC were collected into or cRPMI through the MT Dim (MT^D^), MT Intermediate (MT^I^), and MT Bright (MT^B^) gates described in the Results section.

For Cytospin preparations, ≥50,000 purity-sorted SG^+^/MT^+^ pRBC were centrifuged at 8,000 rpm for 2 min in an Eppendorf centrifuge (Model 5810) at room temperature, and the pellet was resuspended in 100 µL of wash buffer or cRPMI. The suspension was dispensed onto an Epredia Cytofunnel (Fisher Scientific, Cat. No. 59-910-40, USA) with a mounted microscope slide. After centrifugation at 500 rpm (28 g) for 5 min in a Cytospin 4 Cytocentrifuge (Epredia), the pRBC monolayer was fixed with 100% methanol, air-dried for 10 min, and stained with 25% Giemsa solution for 30 min as described above. Microscope images of the stained cells were captured with an Olympus CX41 fitted with a camera using a 100-oil immersion objective lens and Infinity Analyze Software (ACCU-SCOPE version 7.1.0).

### Determination of acetyl CoA carboxylase (*acc*) and skeleton-binding protein 1 (*sbp1*) transcript levels

On the selected follow-up days, 250,000 purity-sorted SG^+^/MT^I^ pRBC were collected from each culture, and cDNA was synthesized using the SuperScript IV Cells Direct Kit (Invitrogen, Cat. No. 11750150) according to the manufacturer’s instructions. DNase I treatment was performed during cell lysis to ensure the synthesized cDNA was free of genomic DNA contamination.

All the primers and probes (File S2) were designed on Benchling (San Francisco, CA; https://www.benchling.com/), and parameters were confirmed using OligoCalc (https://bio.tools/oligocalc) and by BLAST search on PlasmoDB (https://plasmodb.org/plasmo/app). To increase specificity and reduce background fluorescence, all qRT-PCR assays were carried out using the double-quenched hydrolysis probes consisting of 5′ FAM, fluorophores, and a 3' Iowa Black FQ quencher with an additional secondary internal quencher, IDT ZEN (Integrated DNA Technologies, Coralville, IA). The working solution for each primer was 10 pmol/µL, and the hydrolysis probe was constituted to 5 pmol/µL. For a 25 µL PCR reaction mix, 10 µL of PrimeTime Gene Expression Master Mix (IDT, Cat. No. 1055770) was mixed with 2 µL forward primer (final concentration 0.8 µM), 2 µL reverse primer (0.8 µM), 2 µL hydrolysis probe (0.4 µM), 4 µL of PCR water, and 5 µL of the synthesized cDNA solution. The qRT-PCR was performed on a CFX Connect Real-time PCR Detection System (Bio-Rad, USA) for 45 cycles of 95°C for 10 s, 59°C for 30 s, and 72°C for 30 s. After the final cycle, the reaction mix was held at 4°C. Three technical replicates of transcript levels were measured from each of three different biological replicates (3 × 3 = 9 measurements).

Transcription levels of *acc* and *sbp1* were determined on selected assay days relative to day 0 baseline before the first drug exposure. Each gene’s average quantification cycle (Cq) values were calculated from triplicate wells. Due to the known downregulation of the common reference genes for serine tRNA ligase (*sars*) (PF3D7_0717700) and lactate dehydrogenase (*ldh*) (PF3D7_1324900) in parasites exposed to DHA (28), we used a standard curve generated from genomic DNA (gDNA) to quantify the mRNA transcripts of *acc* (PF3D7_1469600) and *sbp1* (PF3D7_0501300) as described by Gray et al. (27). Specifically, gDNA from tightly synchronized ring stage parasites (0–6 h) was serially diluted from 5 to 0.04 ng/μL, confirmed for concentration using a DS-11 series spectrophotometer (DeNovix, Wilmington, USA), and subjected to qRT-PCR as described above. The average Cq values were used to generate a standard curve of gDNA concentration versus average Cq values. This standard curve was then employed to estimate the cDNA concentrations at different time points during and after DHA treatment. Fold change was calculated from a ratio of average Cq values of the treated group on each day to the untreated DMSO control. All statistical analyses were performed using GraphPad Prism 9.3.1 (Dotmatics) using the Mann–Whitney nonparametric test with a *p*-value set at 0.05 for significance.

## Data Availability

Spreadsheets of the primary data collected in this study are provided in the supplemental material.

## SUPPLEMENTAL MATERIAL

File S1 aac.00053-24-s0001.pdf Table S1; Figures S1 to S6.

File S2 aac.00053-24-s0002.xlsx Spreadsheets of data and analyses represented in Fig. 1, 4, and S5.

ASM does not own the copyrights to Supplemental Material that may be linked to, or accessed through, an article. The authors have granted ASM a non-exclusive, world-wide license to publish the Supplemental Material files. Please contact the corresponding author directly for reuse.
