# Supplementary material for: Isolation and characterization of Plasmodium falciparum blood-stage persisters by improved selection protocols using dihydroartemisinin alone
Source: Antimicrob Agents Chemother. 2025 Feb 10;69(3):e00053-24. doi: 10.1128/aac.00053-24 (PMC11881564; doi:10.1128/aac.00053-24)
Supplement: File S1 — Table S1; Figures S1 to S6. [file aac.00053-24-s0001.pdf]

Supplemental Information File 1

Isolation and Characterization of *Plasmodium falciparum* Blood-stage Persisters by Improved Selection Protocols Using Dihydroartemisinin Alone

Daniel Kiboi,[a,b] Juliana M. Sá,[a] Akshaykumar Nayak,[a] Chiara E. Micchelli,[a] Shuchi N. Amin,[a] Alexander G. Burbelo,[a] Sasha A. Abielmona,[a] Brian Xi,[a] Lucia A. Mulei,[a] Noah M. Onchieku,[a] Caroline M. Percopo,[a] Jianbing Mu,[a] and Thomas E. Wellems[a,*]

[a]Laboratory of Malaria and Vector Research, National Institute of Allergy and Infectious Diseases, National Institutes of Health, Bethesda, MD, 20892, USA

[b]Department of Biochemistry, Jomo Kenyatta University of Agriculture and Technology, P.O BOX 62000, 00200, Nairobi, Kenya

[*]To whom correspondence should be addressed: twellems@niaid.nih.gov

Short title: Isolation and study of *P. falciparum* persisters

Contents of this file:

Supplementary Table S1

Supplementary Figures S1–S6

Table S1 Primers and hydrolysis probes used in qRT-PCR analysis of acetyl-CoA carboxylase and skeleton binding protein 1 transcripts.

Gene ID	Gene Name	Forward primer	Hydrolysis probe	Reverse primer
PF3D7_1469600	acetyl-CoA carboxylase	GCA GGA GAA ACT TCA AAA GCG	/56-FAM/TG GTC GCA G/ZEN/T GTT GGT ATT GGT GCT /3IABkFQ/	CCT GTA AGC AAA AGC GAA GAG
PF3D7_0501300	skeleton-binding protein 1	CTC GAC GAT GAC GGT TTT TCA	/56-FAM/AA CGT AGA A/ZEN/T GGC TCA AGA AGC TTT ACT T/3IABkFQ/	TAG GGG ACA TAG ATT CGG CTG

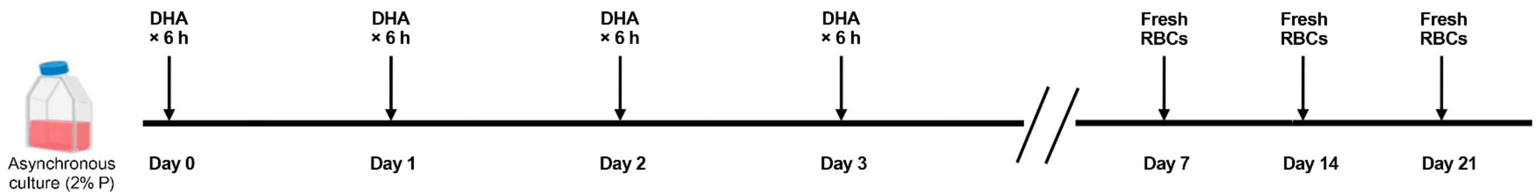

FIG S1 Experimental timeline for the 4DP recrudescence assay with four daily 6-h dihydroartemisinin (DHA) treatments and weekly addition of fresh erythrocytes (RBCs) to counter the possibility of hemolysis. Culture treatments were scheduled at the same time in each 24h interval. Volume of RBC replacement was equivalent to 10% of each culture volume at 5% hematocrit.

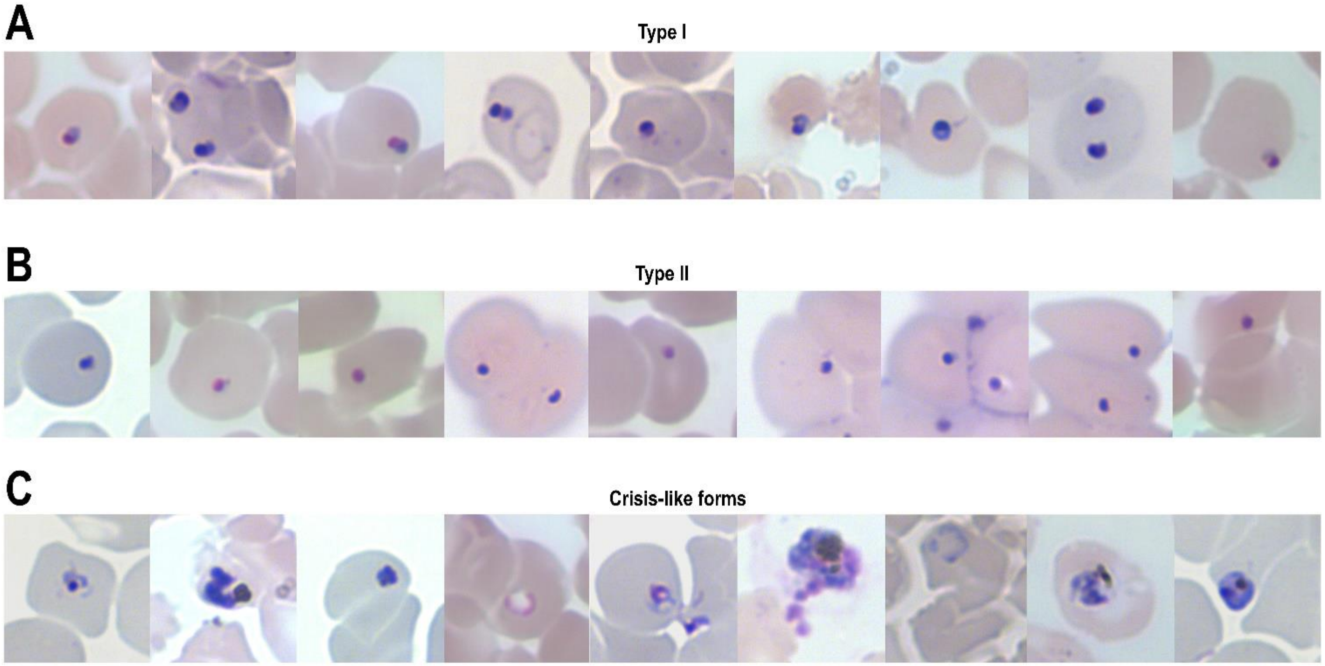

FIG S2 Representative images of Giemsa-stained Type I persisters, Type II persisters, and crisis-like degenerating forms seen after DHA exposure. (A) Type I persisters are characterized by a small, rounded dark magenta-stained nucleus and accompanying blue-stained cytoplasm. (B) Type II have more pyknotic-like or irregular nuclei and lack cytoplasm. (C) Parasites with degenerative changes resemble the *P. falciparum* crisis forms described by Jensen et al. (1).

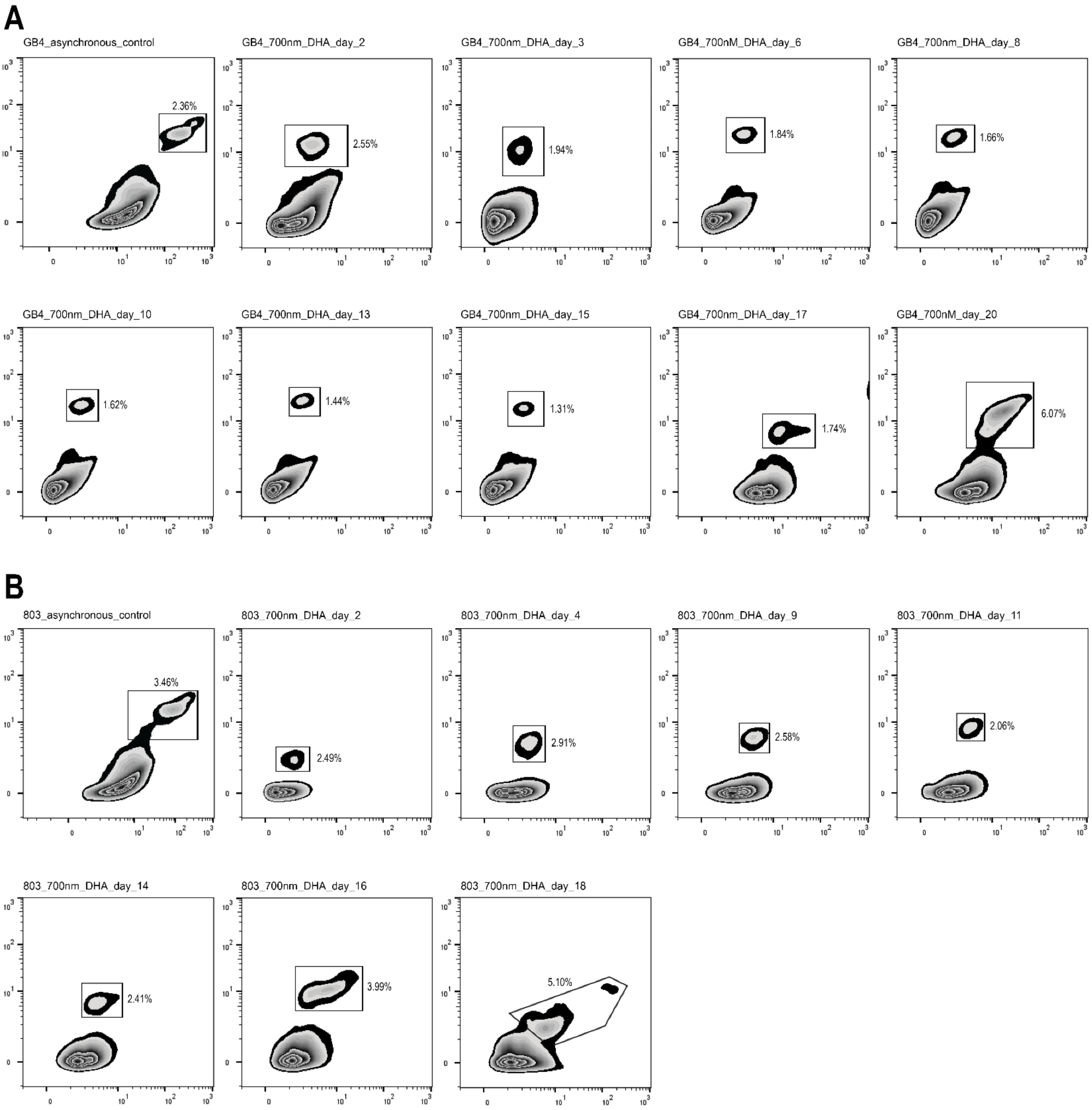

FIG S3 Flow cytometry displays of uninfected and *P. falciparum*-parasitized erythrocytes from 4DP recrudescence experiments using 700 nM DHA. Samples were stained with SYBR Green I (SG) and MitoTracker® Deep Red (MT) and data were obtained from a MACSQuant® Analyzer. The MT and SG fluorescence scales are marked on the horizontal and vertical axes. Estimated parasitemia percentages

are provide next to the gate windows. (A) Upper left panel shows the distribution of brightly MT-stained mature stages from asynchronously growing GB4 parasites before DHA treatment. Panels from the data collected on days 2–15 show the distribution of DHA-treated GB4 persisters, clustered as more dimly fluorescent cells above and slightly to the right of the center of uninfected erythrocytes. On days 17 and 20, the extension of MT^+ distribution to the right indicates the return of parasites reactivated from dormancy. (B) Upper left panel shows the distribution of brightly MT-stained 803 parasites from an asynchronously growing population before DHA treatment. Like the results from GB4 parasites, the panels from data on days 2–11 each show DHA-treated persisters clustered as more dimly fluorescent cells above and slightly to the right of the center of uninfected erythrocytes. Brighter fluorescence signals from recrudescent rings, trophozoites, and schizonts account for the extension of the MT^+/SG^+ distributions to the right of the persisters on days 14–18). The lower position of SG^+ 803 parasites on day 18 may relate to an experimental anomaly, as this variation was not observed in the data collected and shown in maintext Fig. 2.

Sequence: PF3D7_1469600 Range: 1 to 10104

```
                   20                  40                  60                  80                 100                 120                 140                 160                 180                 200
ATGATTAATTTTTTTTTATCATTATTATTGTTTGTTCTATTTTTTGAAAATCTTGTAGTGTCTATAAAATATAGAAATATTCATTATATACACATGCCTAACAATGCTCATAATAAAAACTTTAATGAAAAAGAAAACAGAGAAATATATCATAATGTAAATATATCAGGAGGTAATACAATATATGAAGAACCTAAAAA
TACTAATTAAAAAAAAATAGTAATAATAACAAACAAGATAAAAAACTTTTAGAACATCACAGATATTTTATATCTTTATAAGTAATATATGTGTACGGATTGTTACGAGTATTATTTTTGAAATTACTTTTTCTTTTGTCTCTTTATATAGTATTACATTTATATAGTCCTCCATTATGTTATATACTTCTTGGATTTTT

                  220                 240                 260                 280                 300                 320                 340                 360                 380                 400
TAAGTATATTACTTTGTTTTTATCTAATGGAAAAAAAATCCTACATTCCATTTTTCGAAATAATAATAATAATAATATAACCATATGTAATGAAAATACGGGGAAAAATTCAAACATCAACTTGAAAAATAATTCTGAGAACAATACTAAGAGCAATTCTAAGAATTATTCTAGGGGTTCTTCATCGTGCTTAAATATAC
ATTCATATAATGAAACAAAAATAGATTACCTTTTTTTTAGGATGTAAGGTAAAAAGCTTTATTATTATTATTATTATATTGGTATACATTACTTTTATGCCCCTTTTTAAGTTTGTAGTTGAACTTTTTATTAAGACTCTTGTTATGATTCTCGTTAAGATTCTTAATAAGATCCCCAAGAAGTAGCACGAATTTATATG

                  420                 440                 460                 480                 500                 520                 540                 560                 580                 600
AGAATGTATCCAATACATATTATGATAAGAGAAGAATTAAAAAGGATATTATGAATAAGAAGGTTGAGGAAAATTTGATACATGATGAAAATGAAAATAATATTATTAATGATAAAATATGTTGTGATGAAAAACAAGATTTGGATAAAGAAATTCATGTAGATGAACAAAATGAAAAGAAATCAAACAGTACTTTAAAT
TCTTACATAGGTTATGTATAATACTATTCTCTTCTTAATTTTTCCTATAATACTTATTCTTCCAACTCCTTTTAAACTATGTACTACTTTTACTTTTATTATAATAATTACTATTTTATACAACACTACTTTTTGTTCTAAACCTATTTCTTTAAGTACATCTACTTGTTTTACTTTTCTTTAGTTTGTCATGAAATTTA

                  620                 640                 660                 680                 700                 720                 740                 760                 780                 800
AATGAAAGTTTTATAAGTATATTATCAAATGATAATTCCGAGAAAAAAAAAGATATGGAAGAAAGTAATAATTATTCTAATAATTTTTATCTCAATTCTTTAGAGGAAAACATAATATATCCTACTCAACATGTTGACTGTGATTTTAATGGAAAATGGAAAAGTCATGTAGATGATATTCTAGAGAATGATTCCTACTA
TTACTTTCAAAATATTCATATAATAGTTTACTATTAAGGCTCTTTTTTTTTCTATACCTTCTTTCATTATTAATAAGATTATTAAAAATAGAGTTAAGAAATCTCCTTTTGTATTATATAGGATGAGTTGTACAACTGACACTAAAATTACCTTTTACCTTTTCAGTACATCTACTATAAGATCTCTTACTAAGGATGAT

                  820                 840                 860                 880                 900                 920                 940                 960                 980                1000
TAGTAATATGAATGATAATAGTAATAAATCTTGTATAAGCGATTATGAGTTATTAAAAACGTCTATTTTATTGAATAATACAAAGGATGATTTTCCTCTATATGAAAATTCAAGAAAAAAATATATTAACTACAAGTGGTAAAATAAACAAGAATAAAAACTTAAAAGAAAGAAAAAAAGAAGAATTTAAATAGATTATTTT
ATCATTATACTTACTATTATCATTATTTAGAACATATTCGCTAATACTCAATAATTTTTGCAGATAAAATAACTTATTATGTTTCCTACTAAAAGGAGATATACTTTTAAGTTCTTTTTTATATAATTGATGTTCACCATTTTATTTGTTCTTATTTTTGAATTTTCTTTCTTTTTTTCTTCTTAAATTTATCTAATAAAA

                 1020                1040                1060                1080                1100                1120                1140                1160                1180                1200
ACACTTTAAAGTTAACAAATAATTTTTCATTTAAAAATAGTAAGAATAGAAGAAAATACACAAATAGTTATAATAGTGGATCCTCTACACATTCTCGTTATGTCATGGATAATAAGGAATATATAATTTACCATAATAATAATAATAATAATAATAATAATTACAATAGTTATAGTAATAATTGTAATAATATGTATATT
TGTGAAATTTCAATTGTTTATTAAAAAGTAAATTTTTATCATTCTTATCTTCTTTTATGTGTTTATCAATATTATCACCTAGGAGATGTGTAAGAGCAATACAGTACCTATTATTCCTTATATATTAAATGGTATTATTATTATTATTATTATTATTATTATTAATGTTATCAATATCATTATTAACATTATTATACATATAA

                 1220                1240                1260                1280                1300                1320                1340                1360                1380                1400
CAAGGTAATAAGAAAAAGTATAATAGACTATATTGTAAAAAAAGTGCGAATTCACAAGAAAAGGATTATGATAAGGATATTAATTTAATTGCTTCAGATAAAATTAATAGTGAATTGTCACAAGATGATTTAAAATATAATTCACAGATAATTAATATGCCTAATGACCATTTCAATATTATTACTTCTGATGAAAAAGA
GTTCCATTATTCTTTTTCATATTATCTGATATAACATTTTTTTCACGCTTAAGTGTTCTTTTCCTAATACTATTCCTATAATTAAATTAACGAAGTCTATTTTAATTATCACTTAACAGTGTTCTACTAAATTTTATATTAAGTGTCTATTAATTATACGGATTACTGGTAAAGTTATAATAATGAAGACTACTTTTTCT

                 1420                1440                1460                1480                1500                1520                1540                1560                1580                1600
AAATATAAAAAAAAATGCATATAAAAATTATGTTAATTATATAAATGAAAGAAGATATGGTTATTTTGATTTATTAGAAAAAAAGAATGAAAAAATTATACGCAAATTATTAATAGCTAATAATGGGATGGCAGCCTTAAAATGTATATTATCATTAAAAGATTGGTTATTTAAAAAATTTTATGATGAGAATTTAATTA
TTTATATTTTTTTTTACGTATATTTTTAATACAATTAATATATTTACTTTCTTCTATACCAATAAAACTAAATAATCTTTTTTTCTTACTTTTTTAATATGCGTTTAATAATTATCGATTATTACCCTACCGTCGGAATTTTACATATAATAGTAATTTTCTAACCAATAAATTTTTTAAAATACTACTCTTAAATTAAT

                 1620                1640                1660                1680                1700                1720                1740                1760                1780                1800
AAATTATAGTTATGGCAACTGACGAAGATATTAAAAGTAATGCAAAATATATATCATTAGCAGATAAAGTAATAAAAGTTCCAGGAGGTAAAAATATTCATAATTATGCAAATGTTCCTTTAATTGTTGAGCTAGCCAAAAGTGAGAATGTTGATGCAGTATGGCCTGGATGGGGACATAGTTCAGAAAATCCCTTATTG
TTTAATATCAATACCGTTGACTGCTTCTATAATTTTCATTACGTTTTATATATAGTAATCGTCTATTTCATTATTTTCAAGGTCCTCCATTTTTATAAGTATTAATACGTTTACAAGGAAATTAACAACTCGATCGGTTTTCACTCTTACAACTACGTCATACCGGACCTACCCCTGTATCAAGTCTTTTAGGGAATAAC
                                                                                                                                                  "bc" forward primer
                                                                                                                                                  
                 1820                1840                1860                1880                1900                1920                1940                1960                1980                2000
TCTACTTTGTTAGAAAAAGAAAATATAATATTTATAGGTCCAACTGGTAATGTTATGGAAGCTTTAGGTGATAAAATATCTGCGAATATATTAGCTCAAAGTGTTGAAGTACCTGTGGTAAAATGGAGTGGAGATAATATAAGAATTGATAAATTTGAGAACAATAAAATAAATGACGAACTATATAATAATGCTACAAT
AGATGAAACAATCTTTTTCTTTTATATTATAAATATCCAGGTTGACCATTACAATACCTTCGAAATCCACTATTTTATAGACGCTTATATAATCGAGTTTCACAACTTCATGGACACCATTTTACCTCACCTCTATTATATTCTTAACTATTTAAACTCTTGTTATTTTATTTACTGCTTGATATATTATTACGATGTTA
                           "bc" reverse primer

                 2020                2040                2060                2080                2100                2120                2140                2160                2180                2200
TCATTCATTAGATGATTGTATAAAAGAATGTAAGCGTATTGGTTTTCCTGTTATGATAAAAGCATCACAAGGTGGTGGTGGAAAAGGTATTCGAAAAGTGGAGAATGAATATGAAATAAAAAAAGCATATGAACAAGTACAAAATGAATTACCTAATTCTCCTATATTTTTGATGAAGGTTTGTAATAATGTAAGACATA
AGTAAGTAATCTACTAACATATTTTCTTACATTCGCATAACCAAAAGGACAATACTATTTTCGTAGTGTTCCACCACCACCTTTCCATAAGCTTTTCACCTCTTACTTATACTTTATTTTTTTCGTATACTTGTTCATGTTTTACTTAATGGATTAAGAGGATATAAAAACTACTTCCAAACATTATTACATTCTGTAT

                 2220                2240                2260                2280                2300                2320                2340                2360                2380                2400
TTGAAATACAAGTTGTTGGTGATATGTATGGAAATGTGTGTTCTTTAAGTGGTCGTGATTGTACTACACAAAGAAGATTTCAAAAAATTTTTGAAGAAGGACCACCATCTGTTGTACCATATCCTATATTTCGAGAAATGGAAAAATCATCTATACGATTAACTAAAATGATTAAATATAGAGGTGCTGGAACTATTGAA
AACTTTATGTTCAACAACCACTATACATACCTTTACACACAAGAAATTCACCAGCACTAACATGATGTGTTTCTTCTAAAGTTTTTTAAAAACTTCTTCCTGGTGGTAGACAACATGGTATAGGATATAAAGCTCTTTACCTTTTTAGTAGATATGCTAATTGATTTTACTAATTTATATCTCCACGACCTTGATAACTT

                 2420                2440                2460                2480                2500                2520                2540                2560                2580                2600
TATTTGTATGATCAAATAAATAAAAAATATTTTTTCTTAGAATTAAATCCAAGATTACAAGTAGAACATCCAGTTTCTGAAGGTATAACAAATTGTAATTTAATATCTATCCAATTACAAGTAGCTATGGGAATTCCCCTTCAAAATATAGATGACATTCGAAATTTATATCAAATTGATAAAATAGAAAAAATAAAAAA
ATAAACATACTAGTTTATTTATTTTTTATAAAAAAGAATCTTAATTTAGGTTCTAATGTTCATCTTGTAGGTCAAAGACTTCCATATTGTTTAACATTAAATTATAGATAGGTTAATGTTCATCGATACCCTTAAGGGGAAGTTTTATATCTACTGTAAGCTTTAAATATAGTTTAACTATTTTATCTTTTTTATTTTTT

                 2620                2640                2660                2680                2700                2720                2740                2760                2780                2800
AAAAGATGAACAAAAAAAAGAATTCGAATTAACCGATAATTTGTGTAATGATACTATAAATAAGGATAATATAAATAATGATAATATATATAAAGATAATATAAATAATGATAATATATATAAAGATAATATATATAAAGATAATATAAATAATGATAATATATATAAAGATAATATATATAAAGATAATATAAATAATG
TTTTCTACTTGTTTTTTTTCTTAAGCTTAATTGGCTATTAAACACATTACTATGATATTTATTCCTATTATATTTATTACTATTATATATATTTCTATTATATTTATTACTATTATATATATTTCTATTATATATATTTCTATTATATTTATTACTATTATATATATTTCTATTATATATATTTCTATTATATTTATTAC

                 2820                2840                2860                2880                2900                2920                2940                2960                2980                3000
ATAATATATATAAAGATAATATATATAAAGATAATATATATAATGATAATATATATAAAGATAATATAAATAATGATAATATATACATCATATAGATAATACAACAAATGAACAAAATAATAAGAACTTGTTACATTATAATAATTATAGAAATCAAAATTTATGTAATAATAATTCGATCAAATCCTTATTAAATTATGAT
TATTATATATATTTCTATTATATATATTTCTATTATATATATTACTATTATATATATTTCTATTATATTTATTACTATTATATATGTAGTATATCTATTATGTTGTTTACTTGTTTTATTATTCTTGAACAATGTAATATTATTAATATCTTTAGTTTTAAATACATTATTATTAAGCTAGTTTAGGAATAATTTAATACTA

                 3020                3040                3060                3080                3100                3120                3140                3160                3180                3200
ACAAATGAAAATGTTAATAGAAAGTATAATTTATTGAATGAACACTTTGATTTTTATAATAATAAACCATATATTAAAAATCATGTTATTGCTGCAAGGATTACAGCAGAAAATAGTAATGATAGCTTTAAGCCCACCTCTGGTAATGTAAGAAGAATAAATTTTCAAAACTGGAAAGATGTGTGGGGATATTTTTCTAT
TGTTTACTTTTACAATTATCTTTCATATTAAATAACTTACTTGTGAAACTAAAAATATTATTATTTGGTATATAATTTTTAGTACAATAACGACGTTCCTAATGTCGTCTTTTATCATTACTATCGAAATTCGGGTGGAGACCATTACATTCTTCTTATTTAAAAGTTTTGACCTTTCTACACACCCCTATAAAAAGATA

                 3220                3240                3260                3280                3300                3320                3340                3360                3380                3400
TAATGATGGTTTTGTACATGAGTTTTCAGATTCTCAAATCGGACATATTTTTGCTAAAGGAGAAACAAGAGAAGTAGCAAGAAAAAATTTAATTTTAGCATTAAGAAAGTTACATATTGATGGTGATATAAAAACAGGTACAAAATATTTAGCAAAGATTTTAGAATCTAAAGCATTTATTGACAATAATATTACAACAA
ATTACTACCAAAACATGTACTCAAAAGTCTAAGAGTTTAGCCTGTATAAAAACGATTTCCTCTTTGTTCTCTTCATCGTTCTTTTTTAAATTAAAATCGTAATTCTTTCAATGTATAACTACCACTATATTTTTGTCCATGTTTTATAAATCGTTTCTAAAATCTTAGATTTCGTAAATAACTGTTATTATAATGTTGTT
```

Biotin carboxylase domain

```
             3420                3440                3460                3480                3500                3520                3540                3560                3580                3600
ATTGGCTAGATATAATAATAGAAAAGAAAAAACATGTATTTTATAATACATGTCATATAATATTATTATGTGCTACCATATTTAAACTATTAATATATTTTATGAATGAGAAGGGAAAAGTGGAAGAAAATTTGGATAGAGATGATATAGCTATAAAAAGAGATAAAAATTATGGAAATGTAATAAATAAAAATAATAAC
TAACCGATCTATATTATTATCTTTTCTTTTTTGTACATAAAATATTATGTACAGTATATTATAATAATACACGATGGTATAAATTTGATAATTATATAAAATACTTACTCTTCCCTTTTCACCTTCTTTTAAACCTATCTCTACTATATCGATATTTTTCTCTATTTTTAATACCTTTACATTATTTATTTTTATTATTG

             3620                3640                3660                3680                3700                3720                3740                3760                3780                3800
CATAGTGGTAATATAAATAATAATGGGGAACATATGTGTAAAATGAAAAGTGCATATATATTTGATATGATATTTCAAAATATAAAATATCCTTTTAAAGGATATAATATAGGGGAAAATCTATATCAATTAGAGATAAATGGACAAGAAATAGAAATATCTGCTGAATATGATAAAAATAATAATAAAGTATTTTCTAC
GTATCACCATTATATTTATTATTACCCCTTGTATACACATTTTACTTTTCACGTATATATAAACTATACTATAAAGTTTTATATTTTATAGGAAAATTTCCTATATTATATCCCCTTTTAGATATAGTTAATCTCTATTTACCTGTTCTTTATCTTTATAGACGACTTATACTATTTTTATTATTATTTCATAAAAGATG

             3820                3840                3860                3880                3900                3920                3940                3960                3980                4000
ATTTAATAATCAAACATATATATATGCATGTTCTGAGGATACCTTAGGTATACATATGCAACTAGAGAAAGATAATATTTTCATACCTAATGTTAGGAATCCATATCATTTAATATCTAATACTAATGGGAAAATTGTTAAGTATTTAATAAATGATGGAGAAGAAGTTAAAAAAAATGATGATTATATTGAAGTGGAAG
TAAATTATTAGTTTGTATATATATACGTACAAGACTCCTATGGAATCCATATGTATACGTTGATCTCTTTCTATTATAAAAGTATGGATTACAATCCTTAGGTATAGTAAATTATAGATTATGATTACCCTTTTAACAATTCATAAATTATTTACTACCTCTTCTTCAATTTTTTTTACTACTAATATAACTTCACCTTC

             4020                4040                4060                4080                4100                4120                4140                4160                4180                4200
CTATGAAAATGATTATGACATTTAAATCAACCGAGAGTGGAATATTAAGACATAAATTGTCTGAAGGGACAATTATTAAAATAGGAGATTTATTAGGAATAATAGAAAAAAAAGATAATGATAAAAAACATATAAAACAAGATAATGAAATTCAATATTTTAATGGTCATTTAGATTTGTCAAATAAATATACATATGAA
GATACTTTTACTAATACTGTAAATTTAGTTGGCTCTCACCTTATAATTCTGTATTTAACAGACTTCCCTGTTAATAATTTTATCCTCTAAATAATCCTTATTATCTTTTTTTTCTATTACTATTTTTTGTATATTTTGTTCTATTACTTTAAGTTATAAAATTACCAGTAAATCTAAACAGTTTATTTATATGTATACTT

             4220                4240                4260                4280                4300                4320                4340                4360                4380                4400
TTGATAGATAATCGTACAATATTTCCAAATATTTTAGATGATAATTATAATAAATCTTGTGATAATTCGTATGCCTTTACAGATAATATGTCTTTACAAAACTCTGAAGAACATTATTTGGTAAAAGATGAACAAAAAAAAAAAAAAAAAAAAAAATATTTCTTCCATTTTAAATAATAATATGGTAAGCATAAAAACGGT
AACTATCTATTAGCATGTTATAAAGGTTTATAAAATCTACTATTAATATTATTTAGAACACTATTAAGCATACGGAAATGTCTATTATACAGAAATGTTTTGAGACTTCTTGTAATAAACCATTTTCTACTTGTTTTTTTTTTTTTTTTTTTTTTATAAAGAAGGTAAAATTTATTATTATACCATTCGTATTTTTGCCA

             4420                4440                4460                4480                4500                4520                4540                4560                4580                4600
ATCAAATGATTTAACAGATAATATAAATGTGTTAAGAGCTGAAACATTGAGTGAAGAGGGCTTAAAGGATGAAATATATCATGGTCAAATGTGTGATGATCGAATGTGTGATGATCAAATGTGTGATGATCAAATGTGTGATGATGAAAATGTTGTGAAGAAAAATGATAAAGAACAGAATAAATCACATAAAAATTTAA
TAGTTTACTAAATTGTCTATTATATTTACACAATTCTCGACTTTGTAACTCACTTCTCCCGAATTTCCTACTTTATATAGTACCAGTTTACACACTACTAGCTTACACACTACTAGTTTACACACTACTAGTTTACACACTACTACTTTTACAACACTTCTTTTTACTATTTCTTGTCTTATTTAGTGTATTTTTAAATT

             4620                4640                4660                4680                4700                4720                4740                4760                4780                4800
AAGAAAATAATATGGATGAATGTACTTATGAAGATGATAATTATATATATATGAAAGAAAATCAAAAGAAAAAATTATTTATGAAACAAAATAGAAAGAAAATCTTTCGTTTATTTTCAAATGATAATGAAAAAATAACAACGGCTTTAAATTATTTAAATGATAAATTTCATTGTGTAAAAAATTATTTGTCAAATTTA
TTCTTTTATTATACCTACTTACATGAATACTTCTACTATTAATATATATATACTTTCTTTTAGTTTTCTTTTTTAATAAATACTTTGTTTTATCTTTCTTTTAGAAAGCAAATAAAAGTTTACTATTACTTTTTTATTGTTGCCGAAATTTAATAAATTTACTATTTAAAGTAACACATTTTTTAATAAACAGTTTAAAT

             4820                4840                4860                4880                4900                4920                4940                4960                4980                5000
AATTTTTCTAGTGCAAATTCTGTATCAGATTCTAGTAATTCATCCTATCAAAATAATAAAAACAATAATAATAATAATGACAACAACAACAATAATTATAATAATAATAATAATAATAATAGTAAGAATAAGAAGAAAAATAATAGTGTGCAATACAATTATTCCAATGCTAAATATTCAAATGTTAATATGATACATAA
TTAAAAAGATCACGTTTAAGACATAGTCTAAGATCATTAAGTAGGATAGTTTTATTATTTTTGTTATTATTATTATTACTGTTGTTGTTGTTATTAATATTATTATTATTATTATTATTATTATCATTCTTATTCTTCTTTTTATTATCACACGTTATGTTAATAAGGTTACGATTTATAAGTTTACAATTATACTATGTATT

             5020                5040                5060                5080                5100                5120                5140                5160                5180                5200
GTATGATAAGAAACCATTTGATAAAAGTTATTTAATGAATGAAGTTAACAGTAATAATGTTAATGTGTTAATGATGAAAAATAAAAATAATAGTACGTTCCCCTTGATTGAAAATTTAGAAAATAATATATCTACAGAAATTATGTCTTCTCGAAATACGTCTAATGAAAAAATATTACATAATAATATATCAAAGGATA
CATACTATTCTTTGGTAAACTATTTTCAATAAATTACTTACTTCAATTGTCATTATTACAATTACACAATTACTACTTTTTATTTTTATTATCATGCAAGGGGAACTAACTTTTAAATCTTTTATTATATAGATGTCTTTAATACAGAAGAGCTTTATGCAGATTACTTTTTTATAATGTATTATTATATAGTTTCCTAT

             5220                5240                5260                5280                5300                5320                5340                5360                5380                5400
ATACAATAAGCGAACCAATTTTTAATAATAATTCTTCTGATGAGAGTAATATAAATAATATTACATTTTTTAATAACTTATCAAATAATGGTAGCATAAGAAAAAGAAACAACAATAATAATAGTAGTAGTAGCAATAATAATAATAATAATAATAATAATAATAAAAATAATAATTTTAAACATTCCTATTATATGGAT
TATGTTATTCGCTTGGTTAAAAATTATTATTAAGAAGACTACTCTCATTATATTTATTATAATGTAAAAAATTATTGAATAGTTTATTACCATCGTATTCTTTTTCTTTGTTGTTATTATTATCATCATCATCGTTATTATTATTATTATTATTATTATTATTATTATTTTTATTATTAAAATTTGTAAGGATAATATACCTA

             5420                5440                5460                5480                5500                5520                5540                5560                5580                5600
TATAATAATGATAATATCTATTGGAATCATGTAAAGAATGAAAAAAGCAAATATTTGCTTGACATTCCAATTATGAAAAGAATAGAATTTTTGCTAAAGGGATATGAACAGGATTATGAAAAGTGTTTTGATGAATTAATAAACAAGAAAGATATTAAAAATGTATCAAATTGGTCTGCATATATAATAAACAATATAAA
ATATTATTACTATTATAGATAACCTTAGTACATTTCTTACTTTTTTCGTTTATAAACGAACTGTAAGGTTAATACTTTTCTTATCTTAAAAACGATTTCCCTATACTTGTCCTAATACTTTTCACAAAACTACTTAATTATTTGTTCTTTCTATAATTTTTACATAGTTTAACCAGACGTATATATTATTTGTTATATTT

             5620                5640                5660                5680                5700                5720                5740                5760                5780                5800
TGACATATTAGATACATTCATACAATATAATATTTTATTTTCCAAAAAAGAATTTATTTCAGAAATAGATTTATATGATATATTATATAATAATATAAGGGATAAGAAAAAGCAATATGAAATTATACACGCATATACATACAACGATTTAAGTATAAAGTTTATAGAAAAAATATTAAAATATATATTAAATAATATAA
ACTGTATAATCTATGTAAGTATGTTATATTATAAAATAAAAGGTTTTTTCTTAAATAAAGTCTTTATCTAAATATACTATATAATATATTATTATATTCCCTATTCTTTTTCGTTATACTTTAATATGTGCGTATATGTATGTTGCTAAATTCATATTTCAAATATCTTTTTTATAATTTTATATATAATTTATTATATT

             5820                5840                5860                5880                5900                5920                5940                5960                5980                6000
ATAGTAATTTAGCCTTCGATATAATTTTGGATAAGTTAAAAATATTAGCAGAATTCAAAGGAAAAATCTTTAGAAATATAATTGTATTATCAAGACATATTTTATTTTTATTAGAAGGATTAGAATTAATTGAATATATAAAAATTGCATTAAATTATAATGATAATAAAAATATGAAGAATGGTGGTAAGTTATCAAAT
TATCATTAAATCGGAAGCTATATTAAAACCTATTCAATTTTTATAATCGTCTTAAGTTTCCTTTTTAGAAATCTTTATATTAACATAATAGTTCTGTATAAAATAAAAATAATCTTCCTAATCTTAATTAACTTATATATTTTTAACGTAATTTAATATTACTATTATTTTTATACTTCTTACCACCATTCAATAGTTTA

             6020                6040                6060                6080                6100                6120                6140                6160                6180                6200
AATATGTTACTTTTGTCTAATTATATGAAAAAAAATAATTTAGATTTTTCAAAAATGATAGAATATAAAAATAATAAAAATGATATAGAAATTGTAAATATGTTTTTTAAAGGTCATAGTAGTAATATACATATGTTTGTTCCATCATTAATTAAATCAAATAAAAATTCTATGTTTTTAAAATTTATTTAAATAATTT
TTATACAATGAAAACAGATTAATATACTTTTTTTTATTAAATCTAAAAAGTTTTTACTATCTTATATTTTTATTATTTTTACTATATCTTTAACATTTATACAAAAAATTTCCAGTATCATCATTATATGTATACAAACAAGGTAGTAATTAATTTAGTTTATTTTTAAGATACAAAAATTTTAAAATAAATTTATTAAA

             6220                6240                6260                6280                6300                6320                6340                6360                6380                6400
ATATAAATATTGTAATATTAAATCTATAATGGTAACAAATAATATAATCAAGTTTTCTATAAATAATTCAGAATATACTAATCTATTAATATGGAATGAAAATGATACCATTGATATAAATAAAATTCTTGAGTCAGATATAAAAATAAATAATGATAGATATTTGAATACTGTTCATATAATAAATACAAATAACGAAT
TATATTTATAACATTATAATTTAGATATTACCATTGTTTATTATATTAGTTCAAAAGATATTTATTAAGTCTTATATGATTAGATAATTATACCTTACTTTTACTATGGTAACTATATTTATTTTAAGAACTCAGTCTATATTTTTATTTATTACTATCTATAAACTTATGACAAGTATATTATTTATGTTTATTGCTTA

             6420                6440                6460                6480                6500                6520                6540                6560                6580                6600
TATGTTTACATCCTTCTCATTCGTTTGAAGGAAATATAATAAAAAATAAGATTTTACAAAAATGTAAAAAATTATATATATATAATTATGCAAATAATAAATATGGAGATATATATGAATGGAAAAATGAAGACCTATCAAAAGGATTAGTAGGAAAATATAGTAAAGATGGAAGTATTAATAATATTTTACCTTATGAA
ATACAAATGTAGGAAGAGTAAGCAAACTTCCTTTATATTATTTTTTATTCTAAAATGTTTTTACATTTTTTAATATATATATATTAATACGTTTATTATTTATACCTCTATATATACTTACCTTTTTACTTCTGGATAGTTTTCCTAATCATCCTTTTATATCATTTCTACCTTCATAATTATTATAAAATGGAATACTT

             6620                6640                6660                6680                6700                6720                6740                6760                6780                6800
GAATATATATTTGGTAATGAAAAAGAAATATTGGAATATTATGAATTAAAAAATATTAATCAAGCGATATATGAAAAAACAAAGATATTTTTTGGTATATATAAAAATAATAAAAATGATAATAATATACGTAATAATGTTAATAATAATTATACATCTTTATTTGGACACCGTGTAATAGATTTTAATGAAATAAAAAA
CTTATATATAAACCATTACTTTTTCTTTATAACCTTATAATACTTAATTTTTTATAATTAGTTCGCTATATACTTTTTTGTTTCTATAAAAAACCATATATATTTTTATTATTTTTACTATTATTATATGCATTATTACAATTATTATTAATATGTAGAAATAAACCTGTGGCACATTATCTAAAATTACTTTATTTTTT
```

6820 6840 6860 6880 6900 6920 6940 6960 6980 7000
CACATCAAATGAGTATAATAATTTTGAGGAACATAATTATCAAGATGATAAATTTATATTTAATAAAGATATACATAATATTCTTTTAGAATTAAAGGAATCATTAAATGACATATCACGTGGAAGACTAAATACATTGATAAGAGATAATAAAATAAGTAGTTGTATAATATATCATATTATTGTTGATGATATGATGG
GTGTAGTTTACTCATATTATTAAAACTCCTTGTATTAATAGTTCTACTATTTAAATATAAATTATTTCTATATGTATTATAAGAAAATCTTAATTTCCTTAGTAATTTACTGTATAGTGCACCTTCTGATTTATGTAACTATTCTCTATTATTTTATTCATCAACATATTATATAGTATAATAACAACTACTATACTACC

7020 7040 7060 7080 7100 7120 7140 7160 7180 7200
ATATTGAAACTATAAAAGAGGCATATAAGGTGTTCATGATTAAATATAATGAAATGATATTAGAAAATTATGTCAATAATATATTTATAAAAATATACCGTACTAATAAAAAGTCATGTACTGAAAATGCACCTCAAATTCAATTGGAAAGAATGTTTAAGCTTAACGTGCTTTTAAATAAAGGAGGTGTAAAGAAAGAA
TATAACTTTGATATTTTCTCCGTATATTCCACAAGTACTAATTTATATTACTTTACTATAATCTTTTAATACAGTTATTATATAAATATTTTTATATGGCATGATTATTTTTCAGTACATGACTTTTACGTGGAGTTTAAGTTAACCTTTCTTACAAATTCGAATTGCACGAAAATTTATTTCCTCCACATTTCTTTCTT

7220 7240 7260 7280 7300 7320 7340 7360 7380 7400
AGGACGGATAGATATAATGTGGAAGATGATAATAATAAAAAGGATAATGATAACAAATATAATTGTAATAATAATAATAATGATGATAACAAATATGATTGTAATAATAATTTTTATTATGATAACAAATATGATTGTAATAACAATTTTTATCATGATAACAAATTTAATTGTAATAATAATTATTATCATAATTACCA
TCCTGCCTATCTATATTACACCTTCTACTATTATTATTTTTCCTATTACTATTGTTTATATTAACATTATTATTATTATTACTACTATTGTTTATACTAACATTATTATTAAAAATAATACTATTGTTTATACTAACATTATTGTTAAAAATAGTACTATTGTTTAAATTAACATTATTATTAATAATAGTATTAATGGT

7420 7440 7460 7480 7500 7520 7540 7560 7580 7600
TTTTGTTGAAGAGATTAACCAATTTCCTTCTTTCCAAATTGACACCCTATATATGAAGAGAAAACGCGCTAGAGAAGTAGACACATTATATGCCTATGATTTTATTAATCTTATAAATATATCATTAAACAGATCAAATAAAAACAGAGAAAGTCATAAAATATGTAATTATATTAATAGTATTAAGGAGTTTAAACTAA
AAAACAACTTCTCTAATTGGTTAAAGGAAGAAAGGTTTAACTGTGGGATATATACTTCTCTTTTGCGCGATCTCTTCATCTGTGTAATATACGGATACTAAAATAATTAGAATATTTATATAGTAATTTGTCTAGTTTATTTTTGTCTCTTTCAGTATTTTATACATTAATATAATTATCATAATTCCTCAAATTTGATT

7620 7640 7660 7680 7700 7720 7740 7760 7780 7800
AAAGTGATATGATTTGTTATAATAGTAATAGTGATAATTTAAAGAATCATGCAATGAATATAAAAAGTGCACATATTCCTTTGGAAAAAAAAGAAGAATACCTATTTGAACATTTTGACAATTTAAGTAATTATGAAATAAAAATAAGGAAGTCATTATATTTGTCTGATAAACTAGATATAGGACAGAACAAAAGGAGT
TTTCACTATACTAAACAATATTATCATTATCACTATTAAATTTCTTAGTACGTTACTTATATTTTTCACGTGTATAAGGAAACCTTTTTTTTCTTCTTATGGATAAACTTGTAAAACTGTTAAATTCATTAATACTTTATTTTTATTCCTTCAGTAATATAAACAGACTATTTGATCTATATCCTGTCTTGTTTTCCTCA

7820 7840 7860 7880 7900 7920 7940 7960 7980 8000
GTGGTTGGTTTGTTGTTAAACATAAGAACAGATGAATATGAAGAAGGGAGAGATGTAATATTCATTATTAATGATATAAGTACACAAGGTGGATCATTTAGTATATTTGAAGATGAGCTATTTTATGGTATTTCTAGTTATGCAAGAGAAAAGAAAATTCCACGTATATATATATCATGTAATTCTGGCGCTCGTATAGG
CACCAACCAAACAACAATTTGTATTCTTGTCTACTTATACTTCTTCCCTCTCTACATTATAAGTAATAATTACTATATTCATGTGTTCCACCTAGTAAATCATATAAACTTCTACTCGATAAAATACCATAAAGATCAATACGTTCTCTTTTCTTTTAAGGTGCATATATATATAGTACATTAAGACCGCGAGCATATCC

8020 8040 8060 8080 8100 8120 8140 8160 8180 8200
ATTATATAATTTTTTAATGGATAAAATAAGAATTGAATGGAAAGACGAACAGAAAAAAGAACTAGGTTATAAATATATATATATAACACAAGATGTAAAAGAACAAATAGATAAAGAAGATATTATATTTTTAACTGAAATTATAGAAAATAATGAAAAAAGATATATTATTGATGCTATTGTTGGGAATTTAAAAAATC
TAATATATTAAAAAATTACCTATTTTATTCTTAACTTACCTTTCTGCTTGTCTTTTTTCTTGATCCAATATTTATATATATATATTGTGTTCTACATTTTCTTGTTTATCTATTTCTTCTATAATATAAAAATTGACTTTAATATCTTTTATTACTTTTTTCTATATAATAACTACGATAACAACCCTTAAATTTTTTAG

acc_probe acc_R
8220 8240 8260 8280 8300 8320 8340 8360 8380 8400
CAGTAGGTGTAGAGAATCTCAGAGGAAGTGGATTAATAGCAGGAGAAACTTCAAAAGCGTATGAAGAAATTTTTACATTATCTTATGTTACTGGTCGCAGTGTTGGTATTGGTGCTTATTTAGTTAGACTTGGTAAAAGGACAATTCAAAAAAAAGGCTCTTCGCTTTTGCTTACAGGTTTTAATGCATTGAATAAAATA
GTCATCCACATCTCTTAGAGTCTCCTTCACCTAATTATCGTCCTCTTTGAAGTTTTCGCATACTTCTTTAAAAATGTAATAGAATACAATGACCAGCGTCACAACCATAACCACGAATAAATCAATCTGAACCATTTTCCTGTTAAGTTTTTTTTCCGAGAAGCGAAAACGAATGTCCAAAATTACGTAACTTATTTTAT
acc_F

8420 8440 8460 8480 8500 8520 8540 8560 8580 8600
TTAGGTGAAAATGTATATGTAAGTAATGAACAGTTAGGTGGTGTTAATATTATGATGAGGAATGGTATATCTCAAGTTCAGGTTGAAAGTGATCAAGAAGGTATGGATAAAATTATACAATGGTTATCTTATGTTCCCAGAACTAGTAATGATTATTATGATTTAATACAAAATATATATAAAGAAAATAATAGAAAATT
AATCCACTTTTACATATACATTCATTACTTGTCAATCCACCACAATTATAATACTACTCCTTACCATATAGAGTTCAAGTCCAACTTTCACTAGTTCTTCCATACCTATTTTAATATGTTACCAATAGAATACAAGGGTCTTGATCATTACTAATAATACTAAATTATGTTTTATATATATTTCTTTTATTATCTTTTAA

8620 8640 8660 8680 8700 8720 8740 8760 8780 8800
TCTACAAAATAATAACTTTTTAATAACAAATAAGCAAAAGACTATGAATACATATAACAACCTTTTTATACATAACAATAAAAATGTAAGTAATTCTGTCGATATAAAGGATAACATAAAGAACCAGATAGATACAAATATAAATACGGAAACTAATATTAATGTGCAAGATTTAAATATTATTAAAAAAAATGGGATTA
AGATGTTTTATTATTGAAAAATTATTGTTTATTCGTTTTCTGATACTTATGTATATTGTTGGAAAAATATGTATTGTTATTTTTACATTCATTAAGACAGCTATATTTCCTATTGTATTTCTTGGTCTATCTATGTTTATATTTATGCCTTTGATTATAATTACACGTTCTAAATTTATAATAATTTTTTTTACCCTAAT

8820 8840 8860 8880 8900 8920 8940 8960 8980 9000
ATACAAAATCAAAACAAGATGAAAATGTGTATGAAAAGAAAGATAAAGTAGAAGAAATATATAAAGATCAAAACACATATAGTACAAATAATAGCAAGACCTCTAAACCAAATGATAATTCTATGAGTTCATACAATTTTGAACTTTTACATATAAATGATATGGATTACGATCATATAGATGATTCTAATATTATCGAT
TATGTTTTAGTTTTGTTCTACTTTTACACATACTTTTCTTTCTATTTCATCTTCTTTATATATTTCTAGTTTTGTGTATATCATGTTTATTATCGTTCTGGAGATTTGGTTTACTATTAAGATACTCAAGTATGTTAAAACTTGAAAATGTATATTTACTATACCTAATGCTAGTATATCTACTAAGATTATAATAGCTA

9020 9040 9060 9080 9100 9120 9140 9160 9180 9200
TTGATCAAGGGTACTCAAGAAGAACAGGGTTTCTTAGATAAAAATACCTATTTTGAATATATGAATGAATGGGGAAAAGGTATTATAACTGGTAGAGGAAAATTAGGTTCTATACCTGTTGGATTTATTGCTGTTAATAAAAATTTAGTTACGCAATCTATTCCATGTGATCCAGCATTAAAAACAAAAGCTCAAAAATT
AACTAGTTCCCATGAGTTCTTCTTGTCCCAAAGAATCTATTTTTATGGATAAAACTTATATACTTACTTACCCCTTTTCCATAATATTGACCATCTCCTTTTAATCCAAGATATGGACAACCTAAATAACGACAATTATTTTTAAATCAATGCGTTAGATAAGGTACACTAGGTCGTAATTTTTGTTTTCGAGTTTTTAA

9220 9240 9260 9280 9300 9320 9340 9360 9380 9400
AATACAAGCTCCATGTGTTTTTTTTCCTGATAATTCTTTTAAAACAGCACAATCAATTGAAGATTTTAATAAGGAGAATTTGCCTTTATTTATTTTTGCAAATTGGAGAGGATTTTCAGGTGGATCTATGGATATGTTTTATGGTATCTTAAAATTTGGATCTATGATTGTAAATCAGCTAGTTAATTATAAACACCCTG
TTATGTTCGAGGTACACAAAAAAAAGGACTATTAAGAAAATTTTGTCGTGTTAGTTAACTTCTAAAATTATTCCTCTTAAACGGAAATAAATAAAAACGTTTAACCTCTCCTAAAAGTCCACCTAGATACCTATACAAAATACCATAGAATTTTAAACCTAGATACTAACATTTAGTCGATCAATTAATATTTGTGGGAC

9420 9440 9460 9480 9500 9520 9540 9560 9580 9600
TATTTGTATATATACCTATATCTGCTGAATTGAGAGGTGGATCATGGGTAGTTGTCGATGAAACATTGAATAGCCAAATTATAGAAATGTATGCCGATGTCAATAGTAAAGGGGGAATATTAGAACCACCAGGAATTGTTGAAGTTAAGTTTAGATATCCTGATATTCGAAAATTAATGCACAGTATTGATACTACCATA
ATAAACATATATATGGATATAGACGACTTAACTCTCCACCTAGTACCCATCAACAGCTACTTTGTAACTTATCGGTTTAATATCTTTACATACGGCTACAGTTATCATTTCCCCCTTATAATCTTGGTGGTCCTTAACAACTTCAATTCAAATCTATAGGACTATAAGCTTTTAATTACGTGTCATAACTATGATGGTAT

9620 9640 9660 9680 9700 9720 9740 9760 9780 9800
ATTGCTTTGAATGAAAAAATGGCTAGATGTGAAAATGATGAAGAGAAGAACAATATAAAGAAAGATATAGAAATTAAGGAAAAAGAGTTGTTGCCATATTATTTACAAGTGTGTCATAAATATGCAGATTTACATGACATGTCTACATGTATGAAAGCAAAGGGTGTAATTCGAAAAATTGTCCCATGGAATAAGGCACG
TAACGAAACTTACTTTTTTACCGATCTACACTTTTACTACTTCTCTTCTTGTTATATTTCTTTCTATATCTTTAATTCCTTTTTCTCAACAACGGTATAATAAATGTTCACACAGTATTTATACGTCTAAATGTACTGTACAGATGTACATACTTTCGTTTCCCACATTAAGCTTTTTAACAGGGTACCTTATTCCGTGC

9820 9840 9860 9880 9900 9920 9940 9960 9980 10000
TTCGTTTTTTTATTATCGTCTAATGAGGAGATTATTAATTAACATATTGAGTCGAAAGTATGACAACGCTTTAATCAAAAATGAAGAGATAGAAAATATTTTAAATGACCTTAATAATTCAGAAGATGATGATTATATTGTATGCAATAGAGTTTTCAATAATAATATTTTACGAAATTTAAAATACGATACCAAGGATA
AAGCAAAAAAATAATAGCAGATTACTCCTCTAATAATTAATTGTATAACTCAGCTTTCATACTGTTGCGAAATTAGTTTTTACTTCTCTATCTTTTATAAAATTTACTGGAATTATTAAGTCTTCTACTACTAATATAACATACGTTATCTCAAAAGTTATTATTATAAAATGCTTTAAATTTTATGCTATGGTTCCTAT

10020 10040 10060 10080 10100
TAATATATAACAAAACATTAAATGACTTTTTAAAAATTTTTAAAATGCTATCACAGGAACAAAGGACAGAATTTCTAAATAAAATAAATTCTTATGAAAATTAA
ATTATATATTGTTTTGTAATTTACTGAAAAATTTTTAAAAATTTTACGATAGTGTCCTTGTTTCCTGTCTTAAAGATTTATTTTATTTAAGAATACTTTTAATT

FIG S4 Nucleotide sequence and reading frame of the *P. falciparum* acetyl-CoA carboxylase (*acc*) gene (PF3D7_1469600; NCBI Gene ID: 812246). The forward and reverse primers (acc_F, acc_R) and PCR product detection probe (acc_probe) used in the present study are highlighted in green. The region of the *acc* biotin carboxylase domain is highlighted in red. The forward and reverse primers used by Chen et al. (2) and Peatey et al. (3) to detect PF3D7_1469600 ("*bc*") transcription are highlighted in blue.

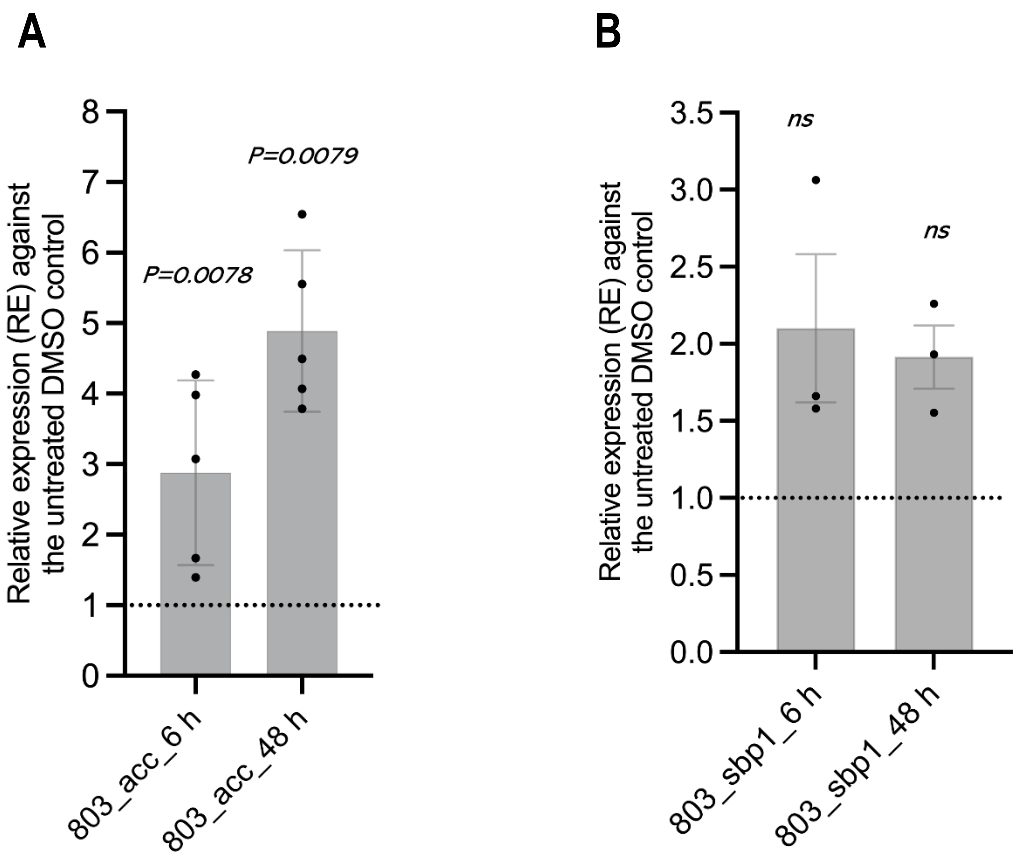

FIG S5 Relative levels of acetyl-CoA carboxylase (*acc*) and skeleton binding protein 1 (*sbp1*) transcripts after a single 6-h pulse exposure of *Plasmodium falciparum* 803 parasites to 700 nM dihydroartemisinin. Results show qRT-PCR measures of (A) reverse-transcribed *acc* transcripts, and (B) reverse-transcribed *sbp1* transcripts at 6-h and 48-h from the start of the pulse. Means and standard deviations are from 5 (*acc*) and 3 (*sbp1*) independent biological replicates.

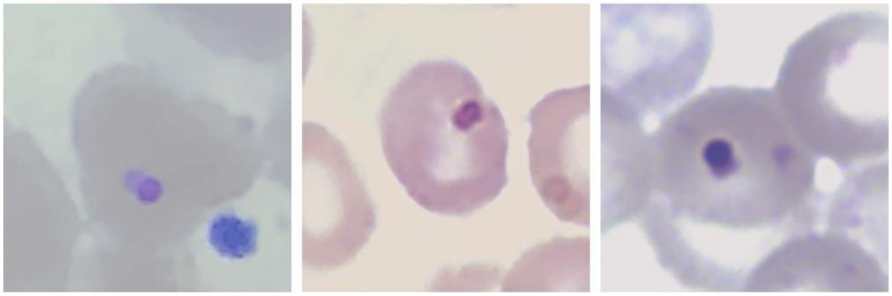

FIG S6 Type I *Plasmodium vivax* persisters observed in the blood of an infected *Saimiri boliviensis* monkey after treatment with a standard 3-day course of daily 4 mg/kg IV artesunate (Sá, J.M. et al., unpublished).

References

1. Jensen JB, Boland MT, Akood M. 1982. Induction of crisis forms in cultured Plasmodium falciparum with human immune serum from Sudan. *Science* **216**:1230-3. doi:10.1126/science.7043736.

2. Chen N, LaCrue AN, Teuscher F, Waters NC, Gatton ML, Kyle DE, Cheng Q. 2014. Fatty acid synthesis and pyruvate metabolism pathways remain active in dihydroartemisinin-induced dormant ring stages of Plasmodium falciparum. *Antimicrob Agents Chemother* **58**:4773-81. doi:10.1128/AAC.02647-14.

3. Peatey C, Chen N, Gresty K, Anderson K, Pickering P, Watts R, Gatton ML, McCarthy J, Cheng Q. 2021. Dormant Plasmodium falciparum parasites in human infections following artesunate therapy. *J Infect Dis* **223**:1631-1638. doi:10.1093/infdis/jiaa562.
